# Angiotensin II-Induced Cardiac Fibrosis: Genomic and Molecular Drivers, Emerging Biomarkers and Therapeutic Perspectives

**DOI:** 10.3390/ijms27219944

**Published:** 2026-11-06

**Authors:** Nancy Lucero Martínez-Rodríguez, Javier Alonzo Campos-González, César López-Camarillo, Oscar Medina-Contreras

**Affiliations:** 1 Unidad de Investigación Epidemiológica en Endocrinología y Nutrición, Hospital Infantil de México Federico Gómez, Mexico City 06720, Mexico; javiercamposmed@gmail.com; 2 Posgrado en Ciencias Genómicas, Universidad Autónoma de la Ciudad de México, Mexico City 03100, Mexico; cesar.lopez@uacm.edu.mx

**Keywords:** angiotensin II, cardiac fibrosis, renin–angiotensin–aldosterone system, myofibroblast, extracellular matrix, biomarkers, heart failure with preserved ejection fraction

## Abstract

Chronically elevated angiotensin II (Ang II) drives adverse cardiac remodelling, converting a reparative response into progressive interstitial and perivascular fibrosis that stiffens the ventricle and precedes overt heart failure. Acting through the angiotensin II type 1 receptor, Ang II generates reactive oxygen species, recruits and polarises immune cells and activates resident fibroblasts, converging on transforming growth factor-β (TGF-β)-dependent and mechanotransductive programmes that drive myofibroblast differentiation and excessive extracellular matrix (ECM) deposition. The magnitude of this response varies between patients: germline variation in renin–angiotensin–aldosterone system (RAAS) genes, loci for heart failure and imaging-defined fibrosis, epigenomic reprogramming of the fibroblast, non-coding RNA networks and metabolic rewiring of collagen-producing cells all modulate the transition from compensated hypertrophy to fibrotic failure. This review integrates these genomic, epigenomic, transcriptomic and metabolomic layers with the signalling architecture of Ang II-induced fibrosis, and reviews the circulating biomarkers—collagen turnover peptides, matricellular and immune mediators, non-coding RNAs and cell-free DNA methylation signatures—that could detect fibrotic activity before remodelling becomes irreversible. We then consider why fibrosis regression under RAAS blockade appears heterogeneous, which emerging targets are mechanistically credible, and how genotype–biomarker–imaging phenotyping could enrich antifibrotic trials. Priorities for ancestrally diverse cohorts and standardised biomarker methodology are identified.

## 1. Introduction

Myocardial fibrosis is the common histological endpoint of nearly every chronic insult to the heart. It is conventionally divided into reparative (replacement) fibrosis, which substitutes collagenous scar for irreversibly lost cardiomyocytes, and reactive fibrosis, which expands the interstitial and perivascular compartments without antecedent cell death [1]. The two processes differ in their cellular choreography and in their reversibility, and the distinction matters clinically: reactive interstitial fibrosis is the dominant lesion in hypertensive, diabetic and neurohormonally driven remodelling, and it is the form most plausibly amenable to pharmacological prevention [2,3].

The clinical burden this lesion underwrites is substantial and rising. Heart failure affects tens of millions of individuals worldwide, and its prevalence continues to increase with population ageing and improved survival from acute coronary syndromes [4,5,6]. Approximately half of prevalent cases are heart failure with preserved ejection fraction (HFpEF), a phenotype in which diastolic dysfunction—driven substantially by interstitial matrix expansion and increased chamber stiffness—rather than contractile failure dominates the physiology [7]. In Latin America, where hypertension prevalence remains high and control rates suboptimal, the contribution of hypertensive heart disease to incident heart failure is correspondingly large [8]. Globally, ischaemic heart disease and stroke remain leading causes of death and disability-adjusted life years lost [9].

Angiotensin II (Ang II) occupies a privileged position in this pathophysiology. It is the effector peptide at which haemodynamic load, systemic neurohormonal activation and locally generated tissue peptide converge, and—critically for a molecular review—its profibrotic actions are not merely a consequence of the pressure it generates. Experimental infusion studies dissociating blood pressure from tissue response demonstrated early that Ang II stimulates the proliferation of perivascular and interstitial fibroblasts through mechanisms independent of blood pressure and aldosterone [10]. Ang II acts directly on cardiac fibroblasts through the angiotensin II type 1 receptor (AT1R) to promote proliferation, resistance to apoptosis and collagen synthesis [11], while simultaneously orchestrating the oxidative and inflammatory environment in which those fibroblasts operate.

Fibrosis is the structural substrate that converts a haemodynamic or neurohormonal insult into permanent loss of function. Interstitial collagen accumulation stiffens the ventricle and impairs diastolic filling, replacement fibrosis after myocyte loss disrupts electrical continuity and supports re-entrant arrhythmia, and perivascular fibrosis increases the diffusion distance for oxygen when demand is greatest. These are not parallel late complications, but one process read out in three ways, and it is the quantity of fibrosis rather than the initiating insult that tracks with mortality, hospitalisation and arrhythmic events across aetiologies. Ang II is the most thoroughly characterised driver of that process, acting on cardiac fibroblasts directly and indirectly through oxidant signalling, sterile inflammation and mechanical feedback to produce a fibrotic programme that persists after the initiating stimulus is withdrawn.

Despite four decades of RAAS pharmacology, a therapeutic gap persists. Angiotensin-converting enzyme (ACE) inhibitors, angiotensin receptor blockers (ARBs), mineralocorticoid receptor antagonists (MRAs) and angiotensin receptor–neprilysin inhibitors all reduce heart failure events, yet no agent is approved with an antifibrotic indication for the heart, and residual risk remains high. Part of the explanation is temporal: myocardial fibrosis is currently detected by cardiac magnetic resonance (CMR) or biopsy at a stage when matrix crosslinking and fibroblast terminal differentiation may already have rendered it substantially irreversible. Part is mechanistic heterogeneity: patients with apparently similar haemodynamic exposure differ markedly in fibrotic response, and that variance is at least partly genomic.

Reviews of this axis have generally treated its layers separately: the signalling literature describes how Ang II activates the fibroblast, the genomic literature describes who is susceptible, and the biomarker literature describes how fibrosis might be detected, with limited traffic between them. The mechanistic and the translational questions are, however, the same question asked at different resolutions—a variant that alters AT1R density, a methylation change that fixes a fibroblast in an activated state, and a circulating propeptide that reports collagen turnover are three observations of one process. This review joins those layers deliberately, and it appraises candidate biomarkers against explicit criteria—analytical validity, incremental value over established measures, and evidence of prospective validation—rather than cataloguing them.

This review therefore organises the Ang II–fibrosis axis along three analytic axes. Section 2 outlines the literature search and reference-verification procedure on which the review rests. Section 3 establishes the pathophysiology—RAAS biology, receptor-level determinants, oxidant signalling, sterile inflammation and fibroblast activation. Section 4 dissects the signalling networks and ECM biology that execute matrix deposition. Section 5, the analytic centre of the review, examines the genomic, epigenomic, transcriptomic and metabolomic layers that determine inter-individual variation in fibrotic response. Section 6 evaluates emerging biomarkers against explicit criteria of analytical validity and incremental clinical value. Section 7 translates these findings into therapeutic and precision-medicine perspectives, and Section 8 and Section 9 state the limitations of the current evidence base and the research priorities that follow from them.

## 2. Literature Search Strategy

A literature search was conducted in Scopus and PubMed/MEDLINE, from database inception to September 2026, covering the genomic and molecular mechanisms by which angiotensin II (Ang II) drives cardiac fibrosis, the determinants of inter-individual variation in that response, and the circulating and imaging biomarkers of adverse cardiac remodelling. Search terms combined Ang II and renin–angiotensin–aldosterone system concepts with fibroblast, extracellular matrix, multi-omic and biomarker terms, cross-referenced against named genes and therapeutic classes; the full term list and the index-specific query structure are given in Supplementary Table S1. In Scopus, the terms were combined as TITLE-ABS-KEY queries restricted to English-language journal articles and reviews and cross-checked against PubMed/MEDLINE, with a further pass restricted to the two most recent publication years to capture recent primary work.

Research and review articles were included because of their contribution to Ang II signalling, cardiac fibroblast and matrix biology, biomarker performance or antifibrotic intervention; extra-cardiac fibrosis literature was admitted only where it establishes a mechanism subsequently demonstrated in the heart. Older work was retained selectively, where it provides the first or definitive description of a mechanism, primary evidence not superseded by later work, or a guideline or consensus document. Every reference was verified against Crossref for resolution of its DOI and agreement of its title with the cited source. This is a narrative review supported by a structured and documented search rather than a systematic review.

## 3. Angiotensin II Biology and the Pathophysiology of Fibrotic Remodelling

### 3.1. The Systemic and Local Renin–Angiotensin–Aldosterone Axis

**Figure 1:**
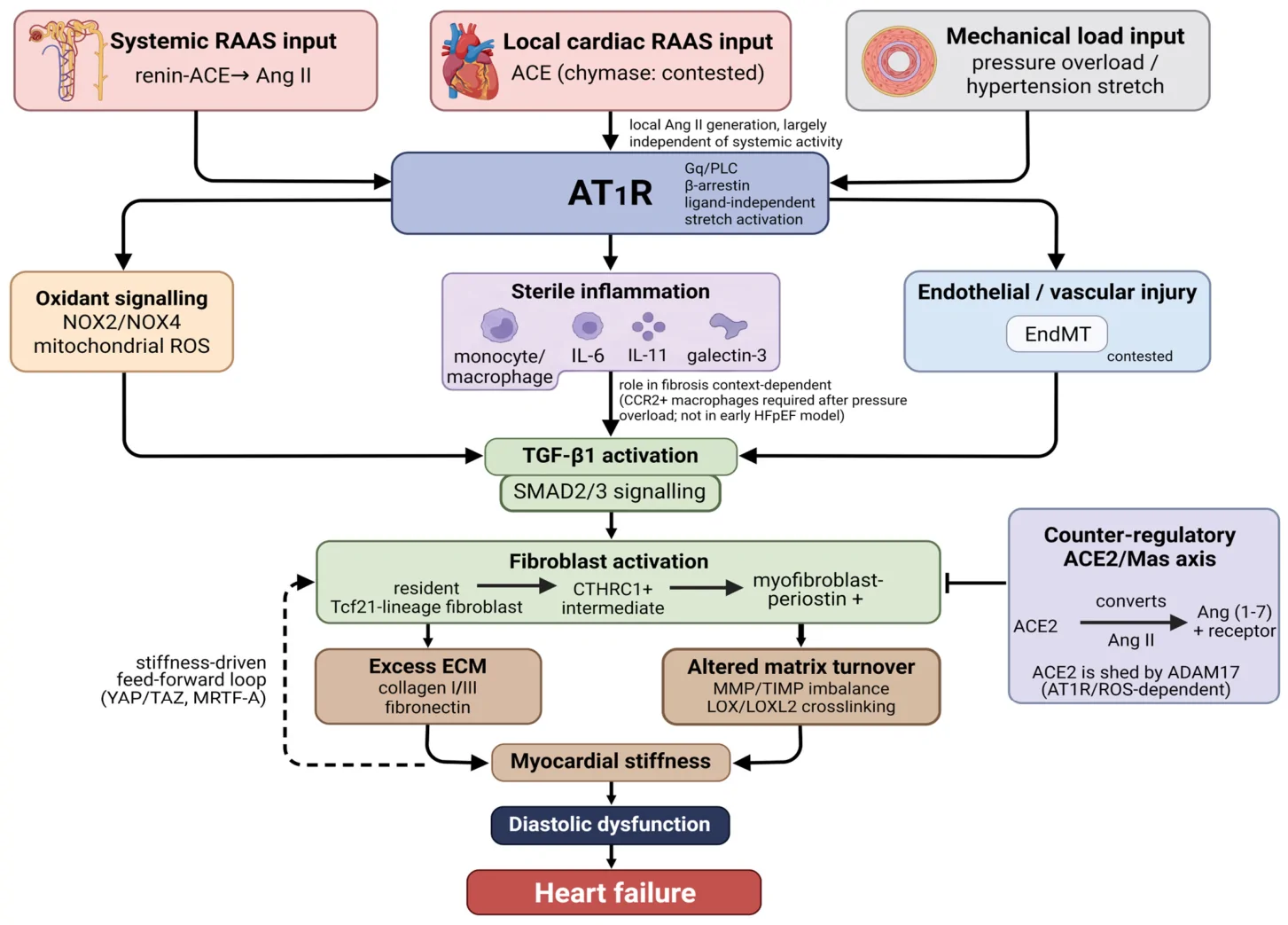
Chronic angiotensin II (Ang II) exposure converts reparative fibroblast activity into progressive fibrosis. Systemic and locally generated Ang II (formed by ACE; a chymase contribution is contested [13,14,15]), together with mechanical load, converge on the AT1 receptor and its Gq/PLC, β-arrestin and stretch-sensitive effectors. Three mutually reinforcing arms—NADPH oxidase- and mitochondria-derived oxidant signalling, monocyte/macrophage-driven sterile inflammation (context-dependent for fibrosis), and endothelial injury with endothelial-to-mesenchymal transition (contested)—converge on TGF-β1 activation and SMAD2/3 signalling, driving resident fibroblasts to the periostin+/CTHRC1+ myofibroblast state. The resulting excess of collagen I/III and the shift in MMP/TIMP balance with increased LOX-mediated crosslinking raise myocardial stiffness, which itself activates mechanosensitive YAP/TAZ and MRTF-A signalling in fibroblasts (dashed line), establishing a self-sustaining feed-forward loop. Abbreviations are defined in the abbreviation list.

The principal elements of this cascade and their downstream consequences are summarised in Figure 1. The classical endocrine cascade—hepatic angiotensinogen cleaved by renal renin to angiotensin I, then converted by pulmonary and endothelial ACE to the octapeptide Ang II—accounts for circulating peptide concentrations but underestimates myocardial exposure. A complete tissue RAAS operates within the heart, with local substrate, processing enzymes and receptors permitting autocrine and paracrine Ang II generation largely independent of systemic activity [12].

The relative contribution of ACE and chymase to local Ang II formation in the human heart is contested. Chymase, a chymotrypsin-like serine protease stored in mast cell granules, generates Ang II from angiotensin I with high efficiency in human cardiac and vascular tissue. Studies of solubilised human ventricular membranes attributed most Ang II-forming activity to chymase [13], whereas intracoronary infusion of labelled Ang I in five heart-transplant recipients showed that enalaprilat reduced fractional conversion by 89%, and a re-examined in vitro assay again attributed approximately 85% of Ang II formation to ACE [14]. Interstitial protease inhibitors have been proposed to suppress chymase in vivo [15], but this rests partly on skin blister fluid as a proxy for cardiac interstitial fluid, and the relative contribution in hypertensive or fibrotic myocardium has not been established. Chymase activity increases in pathological remodelling, and chymase inhibition attenuates fibrosis and functional deterioration in several animal models [16]. If chymase contributes substantially in vivo, this mechanism would provide a coherent explanation for “ACE escape”, the incomplete suppression of tissue Ang II achieved by ACE inhibitors, and would predict that agents acting at or downstream of the receptor have a broader antifibrotic reach than those acting on the converting enzyme; this remains a hypothesis.

Opposing the classical arm is a counter-regulatory axis. ACE2, a carboxypeptidase, cleaves Ang II to angiotensin-(1-7), which signals through the Mas receptor to oppose fibroblast activation, and cleaves angiotensin I to angiotensin-(1-9), an AT2R ligand [12]. Augmentation of the ACE2–Ang-(1-7)–Mas axis attenuates remodelling and fibrosis after myocardial infarction [17], and ACE2-deficient mice show exaggerated cardiac and aortic remodelling in response to Ang II infusion, establishing ACE2 as a determinant rather than a bystander of the fibrotic response [18]. ACE2 availability is itself regulated: the sheddase ADAM17 cleaves ACE2 from the cell surface, reducing local Ang II degradation, and in Ang II-infused mice, AT1R blockade prevented the rise in myocardial ADAM17 activity and the loss of myocardial ACE2, with a reciprocal rise in plasma ACE2 activity [19], a feed-forward loop that would bias the system toward the profibrotic arm; an AT1R-dependent mechanism has also been described in neurons [20]. Because ADAM17 activation also covaried with the pressor response, a direct AT1R effect has not been separated from that of blood pressure [19]. Cardiomyocyte-specific ADAM17 deletion ameliorates left ventricular remodelling in experimental diabetic cardiomyopathy [21]. The ratio of classical to counter-regulatory activity, rather than the absolute Ang II concentration, is therefore a more faithful description of the fibrotic set-point.

Restraint is exerted on the fibroblast itself: Mas signalling truncates AT1R-initiated ERK1/2 output through SHP-1/2, opposes RhoA/ROCK-dependent cytoskeletal tension through nitric oxide–cGMP–protein kinase G, and suppresses fibroblast NOX-derived oxidant signalling [22,23], while angiotensin-(1-7) additionally acts as a β-arrestin-biased AT1R agonist [24]. The axis extends beyond angiotensin-(1-7) [12]. Alamandine signals through MrgD rather than Mas, is antifibrotic in pressure overload, and now has a further family member, alamandine-(1-5) [25]. Angiotensin A is an AT1R agonist that ACE inhibition does not remove and the substrate from which ACE2 generates alamandine; angiotensin III, generated by aminopeptidase A, is a full AT1R agonist but is neutralised by AT1R blockade and is therefore not an escape route [26]; angiotensin IV acts on insulin-regulated aminopeptidase rather than AT1R, so it does not engage the profibrotic receptor at all. Direct cardiac fibrosis evidence exists for alamandine and is lacking for angiotensin A, III and IV.

### 3.2. Receptor-Level Determinants: AT1R and AT2R

Nearly all profibrotic actions of Ang II in the heart are mediated by AT1R, a class A G protein-coupled receptor expressed by cardiomyocytes, fibroblasts, endothelial cells, vascular smooth muscle and infiltrating leukocytes [27]. Canonical Gq/11 coupling activates phospholipase C, generating inositol trisphosphate and diacylglycerol, raising cytosolic calcium and activating protein kinase C, with downstream engagement of mitogen-activated protein kinase cascades and calcineurin–NFAT signalling [11].

Receptor biology is more nuanced than this canonical description. AT1R also signals through β-arrestin-dependent pathways that are kinetically and transcriptionally distinct from G protein signalling, and biased ligands can separate the two outputs [28]. Angiotensin-(1-7) itself behaves as an endogenous β-arrestin-biased AT1R agonist with protective activity in cardiac hypertrophy, illustrating that the two arms of the RAAS interact at the receptor as well as at the peptide level [24].

AT1R can additionally be activated by mechanical stretch in the absence of ligand, through a conformational switch that engages G protein signalling directly [29]. This mechanism is of considerable importance for pressure-overload models and for hypertensive heart disease, because it means that AT1R blockade can attenuate load-induced signalling even when local peptide concentrations are not elevated.

AT2R is expressed at lower density in the adult heart but is upregulated after injury and generally opposes AT1R actions, coupling to phosphatases, nitric oxide and cGMP, and exerting antiproliferative effects. Non-peptide AT2R agonists, of which compound 21 (C21) is the prototype, have shown organ-protective and antifibrotic activity in several experimental settings [30], though cardiac-specific clinical evidence remains limited.

### 3.3. Oxidant Signalling as the Proximal Effector

Ang II is among the most potent physiological activators of NADPH oxidase (NOX) in the cardiovascular system, and NOX-derived reactive oxygen species (ROS) serve as the proximal effector arm that couples receptor occupancy to transcriptional reprogramming. The cardiac NOX isoforms differ markedly in both regulation and functional consequence. NOX2 is agonist-activated, depends on the assembly of cytosolic subunits, and predominates in cardiomyocytes and infiltrating phagocytes, whereas NOX4 is constitutively active, resides in intracellular membranes—including mitochondria and the endoplasmic reticulum—and is controlled chiefly at the level of expression [31]. This distinction is more than semantic: the regulated, target-selective oxidation of reactive cysteine thiols transduces receptor occupancy, whereas oxidative stress refers to a state in which antioxidant capacity is overwhelmed and damage becomes indiscriminate. Because untargeted antioxidants have failed where NOX- and receptor-proximal interventions succeed, Ang II-driven fibrosis belongs to the former category, and “oxidant signalling” is used in that sense throughout.

The functional role of NOX4 is genuinely contested and should not be flattened. Cardiac NOX4 overexpression induces hypertrophy and fibrosis through Akt/mTOR and NF-κB signalling [32], yet NOX4 also mediates adaptive responses: NOX4-deficient mice show worse remodelling under chronic load, an effect attributable to impaired angiogenesis, and both cardiomyocyte and endothelial NOX4 contribute to protection against haemodynamic overload [33,34]. The resolution is probably one of magnitude, compartment and cell type rather than direction, and this unresolved biology is a substantive obstacle to NOX4-directed drug development. The dichotomy tracks cell type and subcellular compartment more closely than it tracks the total amount of enzyme present. In cardiac fibroblasts, NOX4 is induced by TGF-β1 and is required for myofibroblast differentiation and collagen production, placing fibroblast NOX4 on the pro-fibrotic arm in that cell type [35]. In cardiomyocytes the same isoform is bidirectional: it supports load-induced angiogenesis and metabolic adaptation, yet cardiomyocyte NOX4 also shapes resident macrophage-mediated inflammation and diastolic dysfunction, so its net effect depends on the duration and severity of the stress [36]. Compartment matters within the cardiomyocyte as well, since mitochondrially localised oxidant production impairs relaxation through mechanisms distinct from those engaged by endoplasmic-reticulum-associated pools [37]. The practical implication is that a systemically delivered NOX4 inhibitor would act on both arms simultaneously; fibroblast-selective delivery, or a downstream effector restricted to the fibroblast lineage, is a more tractable target than the isoform itself.

ROS do not act indiscriminately. Redox-sensitive targets relevant to fibrosis include the latent TGF-β complex, whose activation is promoted by oxidation; apoptosis signal-regulating kinase 1 and the downstream p38/JNK cascades; and protein tyrosine phosphatases, whose catalytic cysteines are reversibly inactivated by hydrogen peroxide, thereby amplifying tyrosine kinase signalling. Counter-regulation is provided by the KEAP1–NRF2 axis, which coordinates transcription of antioxidant and phase II detoxification genes; NRF2 activation attenuates fibrosis in experimental myocardial injury [38]. The failure of untargeted antioxidant supplementation in cardiovascular outcome trials, set against the efficacy of pathway-specific redox interventions in animal models, argues that compartment-specific rather than global ROS scavenging is the tractable strategy.

### 3.4. Sterile Inflammation and the Immune–Fibroblast Interface

Ang II initiates a sterile inflammatory programme that is now understood to be an obligate intermediate, not an epiphenomenon, in fibrotic remodelling. NF-κB activation downstream of AT1R induces chemokines, adhesion molecules and cytokines that recruit circulating monocytes into the myocardium. Monocyte-derived CCR2+ macrophages are functionally distinct from the resident CCR2− population and are required for adverse remodelling, including interstitial fibrosis, in pressure-overload models (transverse aortic constriction) [39]; in a multi-comorbidity mouse model of early HFpEF, however, CCR2 ablation attenuated cardiomyocyte hypertrophy but not fibrosis or diastolic dysfunction [40], so their role appears context-dependent. Chemokine-mediated regulation of the regulatory T cell compartment further shapes injury severity [41].

Innate immune sensing amplifies this response. Mitochondrial DNA released from stressed cardiomyocytes activates cytosolic nucleic acid sensors and inflammasome signalling in failing hearts [42], and interventions that interrupt the mitochondria–inflammation circuit mitigate experimental HFpEF [43]. Adaptive immunity contributes through CD4+ T cell-derived signals that drive perivascular fibrosis via a KLF10–IL-9 axis [44].

Among cytokines, interleukin-11 (IL-11) has emerged as a particularly proximate effector: it is strongly induced in fibroblasts by TGF-β and is required, downstream of that signal, for fibroblast-to-myofibroblast transition and ECM synthesis across cardiovascular tissues [45]. Its position downstream of the receptor predicts that blockade should spare the broader TGF-β programme, but that selectivity has not been shown directly: the primary study [45] establishes necessity for the fibrotic output, not preservation of the remaining TGF-β functions once IL-11 is neutralised, and other TGF-β-downstream antifibrotic targets have not survived late-phase translation (Section 7.2). Epitranscriptomic control of IL-11 by ALKBH5-dependent m6A modification drives macrophage-to-myofibroblast transition in mice, linking RNA modification to this interface [46]. Interleukin-6 trans-signalling connects the mineralocorticoid arm of the RAAS to the same programme: in low-renin primary aldosteronism and in aldosterone-infused mice, aldosterone induced endothelial IL-6 through the mineralocorticoid receptor/PI3K/Akt/NF-κB pathway and trans-signalling through soluble IL-6 receptor and fibroblast gp130 contributed to fibrosis, which soluble gp130 and eplerenone reduced; the endothelial source was inferred rather than proven, and in patients, fibrosis was inferred from echocardiographic indices rather than histology [47].

Together with the comorbidity-driven inflammatory paradigm proposed for HFpEF, in which systemic inflammation induces coronary microvascular endothelial dysfunction, reduced nitric oxide–cGMP–protein kinase G signalling and consequent cardiomyocyte stiffening and interstitial fibrosis [48], these observations place inflammation upstream of rather than parallel to the matrix response. Platelets contribute to the same interface: deletion of the α-granule chemokine CCL5 attenuated fibrosis, hypertrophy and functional impairment in Ang II-infused mice, with reduced platelet activation and M2 macrophage polarisation [49]. That the interface is genuinely bidirectional is shown by Nr4a1: its deletion prevented interstitial fibrosis and preserved diastolic function through two separable routes, macrophages adopting an anti-inflammatory phenotype and losing much of their capacity to induce fibroblast migration, while Nr4a1-deficient fibroblasts independently failed to convert to myofibroblasts or to overproduce matrix proteins [50].

### 3.5. Fibroblast Activation and Myofibroblast Transition

The identity of the cell that makes the matrix was contested for two decades and is now largely settled. Genetic lineage-tracing established that activated myofibroblasts in the injured heart derive overwhelmingly from resident Tcf21-lineage, PDGFRα+ fibroblasts, with negligible contribution from bone marrow-derived or endothelium-derived cells [51]. Endothelial-to-mesenchymal transition (EndMT), originally reported as a major source of cardiac fibroblasts [52], has been substantially reappraised: rigorous lineage tracing indicates that endothelial cells acquire mesenchymal marker expression without durably becoming matrix-producing fibroblasts in most cardiac contexts [53]. The practical implication is that antifibrotic strategies should target the resident fibroblast pool.

Fibroblast activation proceeds through discrete, transcriptionally definable states rather than as a continuum of a single phenotype. Single-cell analyses after injury identify an early activated fibroblast state, a Postn-expressing α-smooth muscle actin+ myofibroblast state responsible for the bulk of collagen output, and a terminally differentiated “matrifibrocyte” state characteristic of the mature scar, which expresses cartilage- and bone-associated matrix genes [54]. An intermediate CTHRC1+ population marks fibroblasts committed to reparative matrix synthesis [55]. In chronic Ang II exposure, high-resolution transcriptomic profiling of the whole heart has identified the cellular drivers of hypertrophy and fibrosis and the temporal sequence of their activation [56], and in HFpEF models fibroblast activation follows a distinctive pattern that differs from the post-infarction trajectory [57].

Marker specificity remains an acknowledged difficulty: α-smooth muscle actin, periostin, fibroblast activation protein and THBS4 each label overlapping but non-identical activation states, and none is exclusive to the cardiac fibroblast [58]. This matters for biomarker development (Section 6), because a circulating analyte derived from an activation marker inherits the specificity limitations of that marker.

Finally, myofibroblast fate determines whether fibrosis resolves or persists. Deactivation, apoptosis and senescence all remove cells from the matrix-producing pool, and senescent cardiac fibroblasts exert a constraining influence on scar expansion after infarction [59]. Persistence of the activated state—reinforced by matrix stiffness (Section 4.2) and epigenetic fixation (Section 5.4)—is the cellular basis of self-sustaining fibrosis.

## 4. Molecular Signalling Networks Driving Extracellular Matrix Deposition

### 4.1. TGF-β1: The Canonical Convergence Node

**Figure 2:**
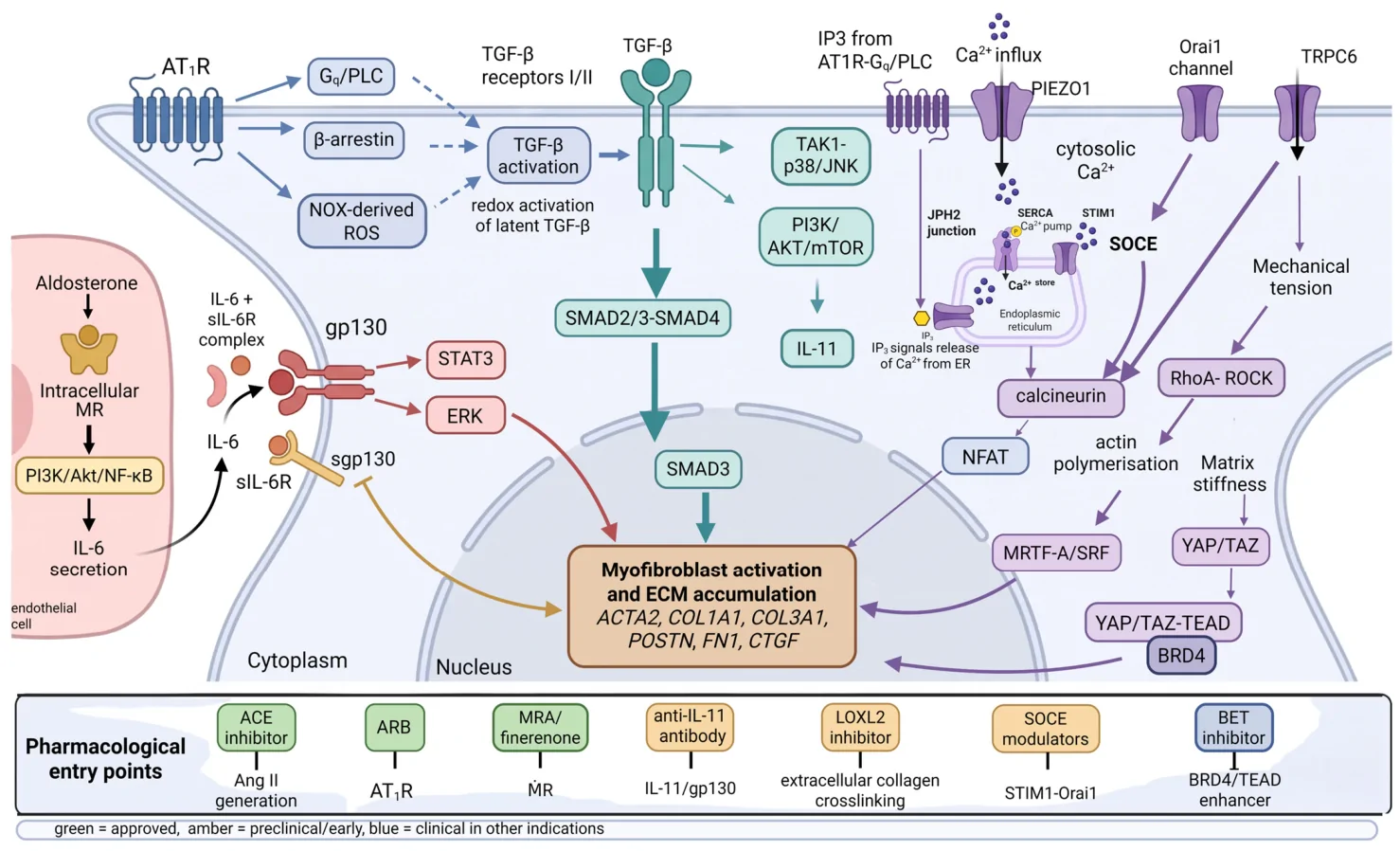
Parallel signalling inputs converge on the myofibroblast transcriptional programme. The principal entry points—AT1R, TGF-β receptors I/II, the mechanosensitive channels PIEZO1 and TRPC6, fibroblast gp130 (engaged by IL-11 and by IL-6–soluble IL-6 receptor complexes), the mineralocorticoid receptor and matrix stiffness—feed distinct proximal cascades that converge on a limited set of nuclear effectors (SMAD3, NFAT, MRTF-A/SRF, STAT3 and YAP/TAZ–TEAD), which control the core pro-fibrotic gene programme (ACTA2, COL1A1, COL3A1, POSTN, FN1, CTGF). This convergence explains why inhibiting a single upstream node is frequently insufficient. Pharmacological entry points are listed beneath the diagram from upstream to downstream, colour-coded by development status. Calcium entry is amplified by release from the endoplasmic reticulum—the fibroblast store, in which release is mediated by the inositol trisphosphate receptor rather than the ryanodine receptor—followed by STIM1–Orai1 store-operated entry at endoplasmic reticulum–plasma membrane junctions organised by junctophilin-2. Because PIEZO1 activation inhibits rather than promotes fibroblast activation, the channel is not a unidirectional target. Solid arrows indicate activation or signal transmission, dashed arrows indicate indirect (redox-dependent) activation of latent TGF-β, and blunt-ended lines indicate inhibition.

TGF-β1 is the principal downstream mediator through which Ang II, oxidant signalling and inflammation converge on the fibroblast; its position relative to the other signalling inputs discussed below is shown in Figure 2. Ang II induces TGF-β1 transcription through AP-1 and related elements, and—more importantly—promotes activation of the large latent complex in which TGF-β1 is stored in the matrix. Latent TGF-β activation is achieved by integrin-mediated mechanical traction (notably αvβ6 and αvβ8), by thrombospondin-1, and by proteolytic and oxidative modification of the latency-associated peptide [60]. Because the latent reservoir is already resident in the ECM, activation rather than synthesis is frequently rate-limiting—a point with direct therapeutic implications.

Canonical signalling proceeds through the type I receptor ALK5 to phosphorylate SMAD2 and SMAD3, which complex with SMAD4 and translocate to the nucleus. Fibroblast-specific deletion studies establish that this axis is both necessary and cell-autonomous: loss of TGF-β–SMAD2/3 signalling specifically in fibroblasts abrogates pressure overload-induced cardiac fibrosis [61], and fibroblast-specific TGF-β signalling likewise mediates fibrosis and dysfunction in obese diabetic mice [62]. SMAD3 is generally the dominant profibrotic effector, while SMAD7 and the SKI/SnoN corepressors provide endogenous braking [63]. Cross-talk with chromatin regulators modulates output: TGF-β1/SMAD3-regulated programmed cell death 5 suppresses post-infarction fibrosis by inhibiting HDAC3 [64].

Non-canonical branches—TAK1–p38/JNK, PI3K/AKT/mTOR, and Rho/ROCK–MRTF-A/SRF—operate in parallel and are responsible for much of the mechanically coupled output. Myocardin-related transcription factor-A (MRTF-A) is retained in the cytoplasm by monomeric G-actin and released upon actin polymerisation, translating cytoskeletal tension into SRF-dependent transcription of contractile and matrix genes; MRTF-A controls myofibroblast activation and fibrosis after myocardial infarction [65].

### 4.2. Mechanotransduction and the Stiffness Feed-Forward Loop

Fibrotic matrix is not an inert product; it is an input. As collagen content and crosslinking increase, tissue elastic modulus rises, and stiffness is itself a potent activating signal for fibroblasts. YAP and TAZ, the transcriptional effectors of the Hippo pathway, are nuclear-localised and active on stiff substrates and cytoplasmic and inactive on soft ones; culture on soft matrix inhibits YAP/TAZ activity, a dependency demonstrated in the context of direct cardiac reprogramming [66].

Mechanical input is transduced in parallel by stretch-activated ion channels. TRPC6, induced downstream of TGF-β and Ang II, is required for myofibroblast transdifferentiation in vivo and acts through a calcineurin-dependent pathway [67]. PIEZO1 is expressed in cardiac fibroblasts and mediates stretch-induced gene expression, positioning mechanosensitive channels as a candidate druggable interface between mechanical load and matrix deposition [68]. Epitranscriptomic control adds a further layer, as YTHDF1-dependent m6A modification of PIEZO2 mRNA regulates cardiac fibrosis [69]. Notably, pharmacological PIEZO1 activation with Yoda1 inhibits rather than promotes TGF-β-induced fibroblast activation through a BRD4-dependent pathway, indicating that mechanosensor signalling is not monotonically profibrotic [70]. Entry through these channels is amplified by release from the endoplasmic reticulum, the fibroblast’s calcium store: inositol trisphosphate receptor-mediated release downstream of the Gq-coupled AT1R—rather than ryanodine receptor-mediated release, which operates in the sarcoplasmic reticulum of the cardiomyocyte—followed by STIM1–Orai1 store-operated entry at endoplasmic reticulum–plasma membrane junctions organised by junctophilin-2, a process required for fibroblast activation and fibrosis in vivo [71]. This machinery is altered in human disease and remains pharmacologically approachable: ventricular fibroblasts from failing human hearts exhibit greater store-operated calcium influx and higher ORAI1 expression than non-failing controls, and their excess collagen secretion is abolished by a selective calcium release-activated channel inhibitor [72]. Calcium-dependent activation therefore presents two targetable nodes—the membrane mechanosensor and the store machinery—both depicted in Figure 2.

Once established, this loop can sustain fibrosis after the initiating neurohormonal stimulus has been pharmacologically suppressed, which is the strongest mechanistic argument for intervening early—before the mechanical feed-forward becomes autonomous.

### 4.3. Parallel and Modulatory Pathways

CCN2/CTGF. CCN2 is induced by TGF-β and Ang II and functions as an autocrine amplifier of fibroblast activation [73]. Its role is context-dependent rather than obligatory: cardiac CTGF knockout does not prevent hypertrophy or fibrosis under chronic pressure overload, indicating redundancy in the amplification layer [74]. Elevated circulating CCN2 nonetheless tracks with fibrotic burden and outcome in high-risk populations.

Aldosterone and the mineralocorticoid receptor. Aldosterone induces cardiac fibrosis directly, independent of its renal actions [75], with IL-6 trans-signalling as one effector, demonstrated independently of Ang II and set out in Section 3.4 [47]. Because Ang II stimulates adrenal aldosterone release and because “aldosterone breakthrough” occurs during chronic ACE inhibition, the mineralocorticoid arm is best regarded as an integral component of RAAS-driven fibrosis rather than a separate pathway.

Wnt and related developmental pathways. Wnt/β-catenin signalling is reactivated in the injured heart and promotes fibroblast activation; multiple upstream regulators converge on this axis in experimental cardiac fibrosis [76,77], and it is embedded in the broader signalling network mapped for myocardial injury [78].

Galectin-3. Galectin-3 is secreted principally by activated macrophages and acts on fibroblasts to promote proliferation and collagen production, positioning it simultaneously as a mediator and, as discussed in Section 6, as a measurable biomarker [79]. This dual role is analytically useful but clinically ambiguous, since a mediator-derived biomarker reports pathway activity rather than tissue fibrosis per se.

Matricellular proteins. Osteopontin, tenascin-C, thrombospondins and SPARC modulate cell–matrix interaction without primary structural function [80]. Osteopontin is required for the full fibrotic response to Ang II in the intact heart [81] and is mechanistically linked to Ang II-driven miR-21 transcription through AP-1 [82].

### 4.4. Extracellular Matrix Composition, Turnover and Crosslinking

The fibrotic myocardial matrix differs from its physiological counterpart in composition, organisation and mechanical behaviour. Type I collagen, which confers tensile stiffness, increases disproportionately relative to the more compliant type III, raising the collagen I/III ratio and with it chamber stiffness [83]. Fibronectin splice variants, proteoglycans and matricellular proteins are concurrently remodelled [80].

Net matrix accumulation is determined not by synthesis alone but by the balance between matrix metalloproteinases (MMPs) and their tissue inhibitors (TIMPs). Ang II, cytokines and ROS all modulate MMP expression and activity, and the stoichiometry of MMP to TIMP determines whether increased collagen synthesis translates into accumulation [84].

Crosslinking is the step that converts reversible matrix expansion into durable, mechanically consequential fibrosis. Lysyl oxidase (LOX) and LOX-like 2 (LOXL2) catalyse covalent crosslink formation between collagen fibrils, increasing both stiffness and resistance to proteolytic degradation; LOXL2 is specifically implicated in cardiac interstitial fibrosis [85]. Pharmacological LOXL2 inhibition reduces crosslinking and fibrosis in experimental models, and small-molecule inhibitors with demonstrated target engagement have entered development [86]. Non-enzymatic crosslinking by advanced glycation end products contributes an additional, largely irreversible component in diabetes and ageing [87]. The distinction between crosslinked and non-crosslinked matrix is central to the reversibility question addressed in Section 7. Table 1 summarises the principal signalling nodes discussed above, their experimental support and their therapeutic tractability.

## 5. Genomic, Epigenomic and Metabolomic Drivers of Inter-Individual Variation

**Figure 3:**
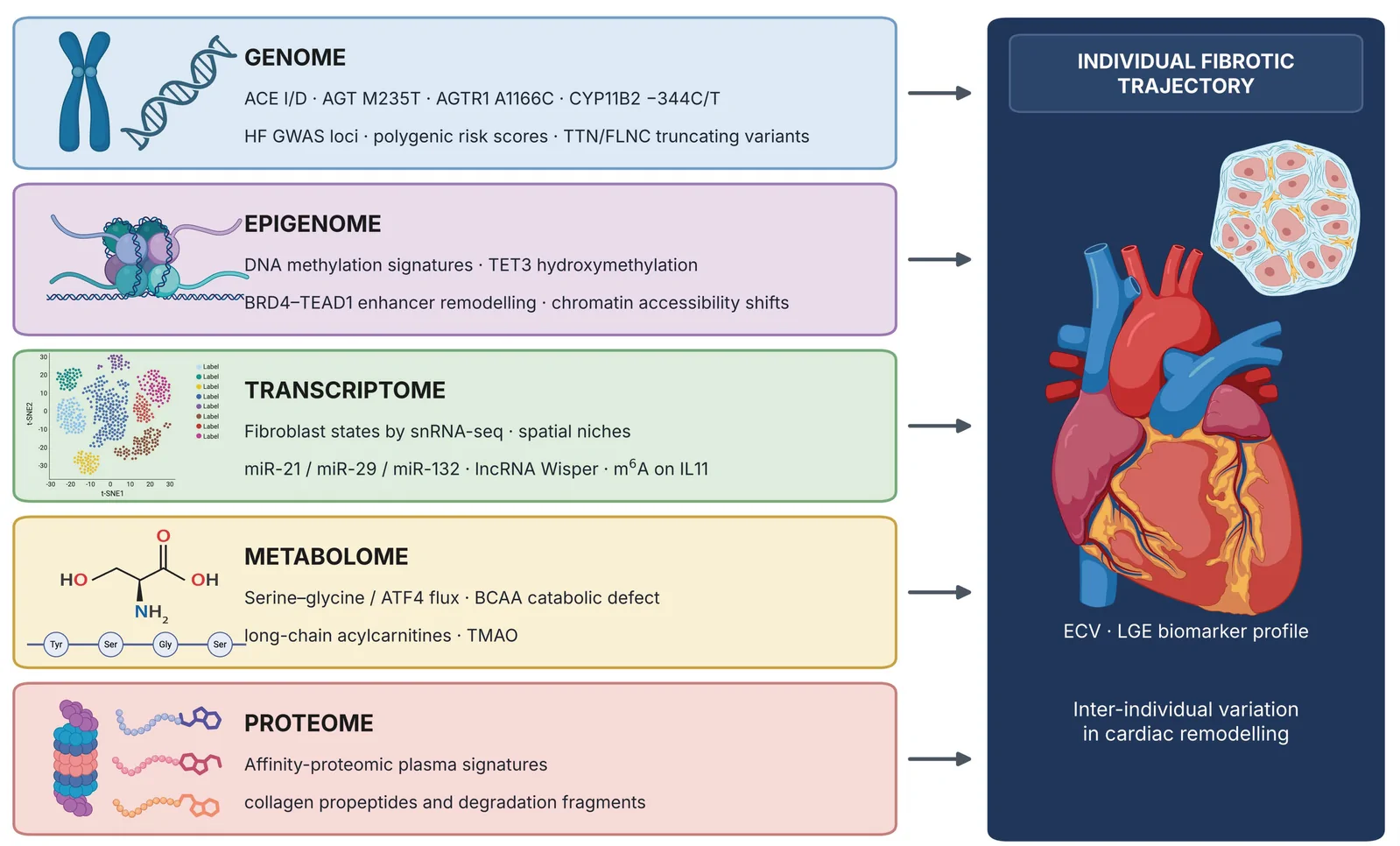
Molecular layers generating inter-individual variation in the fibrotic response to angiotensin II. Germline variation in RAAS genes and genome-wide heart failure loci set a baseline of susceptibility; epigenomic reprogramming, transcriptional and non-coding RNA networks, metabolic rewiring and the circulating proteome each add further variance, and together they shape the individual fibrotic trajectory, read out clinically as the extracellular volume and late gadolinium enhancement imaging phenotype (**right**). Most of the supporting evidence derives from European-ancestry cohorts, which constrains transferability of polygenic estimates to other populations, including Latin American populations (Section 5.2).

The molecular layers surveyed in this section are summarised in Figure 3. The mechanisms described above are shared by all mammalian hearts; the variance they produce is not. Two patients with comparable blood pressure burden and comparable duration of exposure can differ several-fold in extracellular volume fraction and in the rate at which diastolic dysfunction progresses. This section examines the molecular layers that generate that variance; the same layers have recently been surveyed together for cardiac fibrosis as a whole, with emphasis on how their integration exposes mechanisms invisible to any single platform [88].

### 5.1. Germline Variation in RAAS Genes

The candidate-gene era focused intensively on RAAS polymorphisms, and the resulting literature requires careful interpretation: most individual studies were underpowered, population-stratified and analysed without genome-wide correction, and replication has been inconsistent.

The ACE insertion/deletion (I/D) polymorphism in intron 16 is the most extensively studied. A meta-analysis of 17 case-control studies (5576 participants) found no association between the I/D polymorphism and heart failure risk under any of four genetic models [89]. Direct evidence linking the I/D polymorphism to imaging- or biopsy-defined myocardial fibrosis is sparser still, and the absence of a pooled association even with clinical heart failure suggests that any genotype effect on collagen deposition is small and context-dependent rather than a dominant driver. In a Mexican cohort of 239 hypertensive and 371 non-hypertensive individuals, the I/D polymorphism was likewise not associated with hypertension, whereas six other ACE promoter and intronic variants were, and the GGATG haplotype carried both an increased risk of hypertension (OR 1.41) and significantly higher serum ACE concentrations than the AAGCA haplotype, linking genotype to measurable enzyme level in an admixed population [90]. In a separate Mexican series of 236 patients undergoing coronary stenting and 455 controls, ACE haplotypes were associated with coronary artery disease (AAAGCA, OR 1.31, 95% CI 1.04–1.66) but not with in-stent restenosis [91].

Angiotensinogen (AGT) M235T is in tight linkage disequilibrium with a promoter variant and is associated with higher plasma angiotensinogen concentrations; meta-analyses support association with plasma angiotensinogen and with hypertension, most clearly in White participants, but explicitly not with ischaemic heart disease or myocardial infarction in either ethnic group, with small per-allele effects [92,93]. Contemporary work continues to examine composite RAAS genotype in relation to blood pressure control [94]. Aldosterone synthase (CYP11B2) −344C/T has been associated with left ventricular hypertrophy in some cohorts [95] and with left ventricular structural indices in meta-analysis [96], though association with hard cardiovascular events is inconsistent [97]. Angiotensin II type 1 receptor (AGTR1) variation has been examined in the same Mexican population, where the rs5182 CC genotype was associated with hypertension (OR 1.83) and a two-variant haplotype with a smaller increase in risk; the A1166C variant that dominates this literature was not among those genotyped [98].

The appropriate summary is that RAAS candidate variants exert small, population-dependent effects on intermediate phenotypes, are inadequate as standalone predictors, and are most defensibly used either as components of pathway-level scores or as pharmacogenomic modifiers (Section 7.4). Two recent analyses sharpen this picture in ways that bear directly on ancestry. A genome-wide association study of circulating angiotensinogen identified 115 associated variants, with the lead signal differing by group—AGT rs4762 (G > A) in White and Hispanic participants, rs5050 (T > G) in Black participants and rs16852311 (G > C) in Chinese participants—and mediation analysis indicated that rs4762 and rs5050 raise systolic and diastolic pressure and the odds of hypertension indirectly, through their effect on angiotensinogen concentration, with opposing direct effects that can mask the total association [99]. Separately, a discovery analysis of resistant hypertension found 24 associated variants, 15 of whose risk-increasing alleles were associated with lower serum potassium and higher hyperaldosteronism risk; only aldosterone antagonists raised potassium in resistant cases, and Mendelian randomisation was consistent with resistant hypertension being largely a manifestation of genetically determined hyperaldosteronism [100]. The clinical corollary is that pathway position, not just pathway membership, determines which patients are mineralocorticoid-driven.

### 5.2. Genome-Wide Discovery: From Heart Failure Loci to Fibrosis Loci

Genome-wide association studies have transformed the resolution of this question by removing the candidate-gene prior. Large multi-ancestry analyses have identified numerous loci for heart failure and, importantly, have begun to partition them by subtype, distinguishing signals acting through coronary disease or atrial fibrillation from those acting on the myocardium directly [101]. Integration with Mendelian randomisation and plasma proteomics has been used to nominate causal proteins and prioritise druggable targets within these loci [102]. GWAS of dilated cardiomyopathy has identified the loci influencing myocardial resilience, defining genetic modifiers of the response to insult rather than the insult itself [103].

The most directly relevant advance for this review is genome-wide analysis of myocardial interstitial fibrosis quantified from CMR native T1 in large imaging-genetics cohorts, which has identified loci associated with the fibrotic phenotype itself rather than with its clinical sequelae [104]. This design—imaging-derived quantitative endophenotype as the trait—is the template for future discovery, because it measures the process rather than an endpoint several causal steps downstream.

Rare variation contributes on a different scale. CMR comparison of FLNC and TTN truncating variant carriers demonstrates genotype-specific remodelling signatures in these arrhythmogenic, fibrosis-rich cardiomyopathies [105]. Single-cell analysis of human hearts carrying pathogenic cardiomyopathy variants has shown that these variants alter cellular composition and cell-state distribution, providing a mechanistic bridge between genotype and tissue-level fibrosis [106].

Polygenic risk scores aggregate this variation into an individual-level estimate. PRS for heart failure predict incident disease independently of clinical risk factors [107], but their transportability across ancestries is limited by differences in linkage disequilibrium structure and allele frequency [108,109]. This limitation is not abstract for the population served by Latin American centres: blood pressure genetic risk scores derived in European-ancestry cohorts show attenuated performance in admixed Hispanic samples [110]. Any clinical deployment of fibrosis-related PRS in admixed populations requires ancestry-aware derivation or recalibration.

Mendelian randomisation provides a complementary, confounding-resistant approach to causal inference. MR analyses have supported causal effects of blood pressure lowering through specific antihypertensive drug targets on heart failure risk [111], and have been used to examine clonal haematopoiesis in relation to cardiac remodelling phenotypes [112].

Non-germline and ageing-related sources of variation. Not all heritable-looking variation in fibrotic response is germline. Clonal haematopoiesis of indeterminate potential—somatic expansion of mutant haematopoietic clones, most often carrying DNMT3A or TET2 lesions—alters the inflammatory environment of the myocardium: DNMT3A-driver clones promote cardiac fibrosis through paracrine activation of cardiac fibroblasts [113], mosaic loss of the Y chromosome in leukocytes causes fibrosis and increases heart failure mortality in men [114], and clonal haematopoiesis is enriched in clinical and experimental heart failure with preserved ejection fraction [115]. Biological ageing contributes a second non-germline axis, with systemic ageing processes converging on the same fibrotic effectors [116] and environmental and genetic predictors of cardiovascular ageing now separable at population scale [117]. Sex modifies the response to the stimulus itself, Ang II infusion producing sex-specific ventricular remodelling in aged animals [118], which, together with the human associations discussed in Section 8, argues for sex-stratified rather than sex-adjusted analysis of fibrotic endpoints.

### 5.3. Transcriptomic and Single-Cell Architecture of the Fibrotic Myocardium

Bulk transcriptomics of failing myocardium reliably identifies matrix and TGF-β pathway enrichment but cannot distinguish increased per-cell expression from increased fibroblast abundance—a confound that single-cell and single-nucleus approaches resolve directly. The adult human heart cell atlas defined the major cardiac cell populations, their regional heterogeneity and their transcriptional states, providing the reference framework against which diseased hearts are now compared [119]. Subsequent work has extended this to disease states and has been reviewed comprehensively for cardiovascular application [120], and isoform-resolved atlases now add alternative splicing as a further axis of fibroblast state definition [121].

Spatial transcriptomics addresses the limitation intrinsic to dissociation-based methods—loss of tissue context—and is essential for fibrosis, where the relevant biology is the spatial relationship between fibroblasts, immune cells, vessels and cardiomyocytes. Integrated single-cell and spatial analysis of remodelling myocardium has identified macrophage–fibroblast signalling niches, including TREM2-associated populations [122], and spatial immune profiling of human infarcted myocardium has mapped the geography of the immune–fibroblast interface [123]. Chromatin accessibility profiling at single-cell resolution complements this by identifying the transcription factor motifs that define fibroblast activation states, including in diabetic cardiomyopathy where combined single-cell ATAC and transcriptomic analysis has defined disease-specific fibroblast programmes [124]. Integration of three-dimensional biomechanics with epigenomics has begun to characterise atypical fibroblast states arising under specific mechanical conditions [125].

Two caveats apply throughout. First, single-nucleus methods are generally required for adult human myocardium because intact cardiomyocytes are refractory to standard dissociation, and nuclear and cytoplasmic transcriptomes differ systematically. Second, most human datasets derive from explanted end-stage hearts or from donors declined for transplantation, which means the early, potentially reversible stages of Ang II-driven fibrosis are systematically under-sampled.

Human evidence published since has extended the map in several directions. Single-nucleus sequencing across non-failing, dilated and ischaemic cardiomyopathy hearts, including the ischaemic scar, found that the resident fibroblast states occupying perivascular and interstitial niches in non-failing myocardium are markedly depleted in heart failure as they give rise to disease states, that activation ligands and transcriptional programmes differ between scar and interstitial fibrosis and between ischaemic and non-ischaemic interstitial fibrosis, and that trajectories diverge according to the resident state from which they originate [126,127]. In heart failure with preserved ejection fraction, single-nucleus profiling of pooled myocardial biopsies found fibroblasts to be the most transcriptionally perturbed population, exceeding cardiomyocytes, with immune activation, metabolic and protein quality-control pathways shared preferentially between the two [128]. Spatially resolved whole-transcriptome profiling has begun to anchor these states to histological features, while documenting the platform’s unequal compartmentalisation accuracy across cell types, high for cardiomyocytes and limited for fibroblasts and endothelium [129]. Integrated proteomic, tissue-transcriptomic and genetic analysis of pressure overload in aortic stenosis has additionally prioritised circulating mediators that associate with interstitial fibrosis and with clinical outcome in independent cohorts [130].

Computational limitations bear directly on what these datasets can establish. Fibroblast activation states are defined by clustering, and cluster boundaries are not observer-independent: resolution choice, normalisation and the method used to integrate donors and batches all change the number and composition of the states recovered, and systematic benchmarking of atlas-level integration shows that no single method dominates across data types, with approaches that maximise batch mixing tending to erode biological structure [131]. Transitional populations are the most vulnerable to this. A CTHRC1+ or intermediate activation state may represent a genuine continuum, an artefact of doublets formed between a fibroblast and an adjacent myeloid or endothelial cell, or an unstable cluster recovered by one pipeline and not another; dedicated benchmarking of doublet-detection methods exists for the second possibility and should be applied before a transitional state is interpreted biologically [132]. Ordering cells along a pseudotemporal trajectory does not settle the question either, since trajectory-inference methods disagree substantially on topology for the same input and their accuracy depends on the underlying branching structure [133]. Multi-omic integration adds a further layer, because transcriptomic, chromatin-accessibility and spatial measurements are typically made on different cells from different samples, so integration rests on anchoring assumptions whose violation is difficult to detect from the integrated object alone [134]. For fibrosis the practical consequence is that a putative transitional state should be corroborated by at least one orthogonal measurement—spatial localisation, protein-level marker detection or lineage tracing—before it is treated as a biological entity rather than a clustering result. The activation trajectory recovered from human myocardium is largely shared across aetiologies, and no human dataset contrasts Ang II-driven remodelling with a distinct stimulus at matched fibrotic burden; these data therefore define the fibroblast states through which Ang II acts, not a state specific to it.

### 5.4. Epigenomic Reprogramming: How the Fibroblast Remembers

Fibrosis frequently persists after the initiating stimulus is withdrawn, and the leading explanation is epigenetic fixation of the activated fibroblast state; the DNA methylation, histone and chromatin accessibility layers of that reprogramming are considered in turn below.

DNA methylation. Differential methylation in failing myocardium associates with altered metabolic and structural gene expression [135], and cardiomyocyte-specific methylation signatures have been defined in human heart failure [136]. The most mechanistically instructive evidence comes from renal fibrosis, where hypermethylation-dependent silencing of the Ras-GTPase-activating protein RASAL1 maintains fibroblast activation, and where TET3-mediated hydroxymethylation or pharmacological demethylation with hydralazine reverses that silencing and attenuates fibrosis [137,138]. Whether an equivalent methylation-locked circuit operates in the cardiac fibroblast is an open and testable question; the mechanism is cited here as a paradigm, not as established cardiac biology.

Histone modification and bromodomain readers. BRD4, a BET-family acetyl-lysine reader, occupies enhancers that are dynamically remodelled during fibroblast activation and is required for the transition to the pathological state [139]. TEAD1 induces fibroblast remodelling through a BRD4/Wnt4 pathway, linking mechanosensitive transcription factors to chromatin readers [140], and BRD4 inhibition rewires cardiac macrophages toward a protective, low-MHC class II phenotype, indicating that BET inhibition acts on multiple cell types in the fibrotic niche [141]. Clinically, the oral BET inhibitor apabetalone has been evaluated in cardiovascular outcome trials, with pooled analyses suggesting reduction in major adverse cardiovascular events [142]; whether any of this benefit is attributable to an antifibrotic mechanism has not been established. Histone deacetylases also participate, as illustrated by HDAC3 regulation downstream of TGF-β1/SMAD3 [64].

RNA modification. The epitranscriptome adds a further regulatory layer: m6A-dependent control of IL-11 governs macrophage-to-myofibroblast transition [46] and YTHDF1-dependent m6A regulation of PIEZO2 modulates cardiac fibrosis [69]. The same reader governs a transcription-factor node, since fibroblast-specific Ythdf1 deletion and Runx1 silencing both reduced experimental fibrosis and m6A methylation of RUNX1 enhanced cytoskeletal remodelling—evidence that modification state, not transcript abundance alone, determines transcription-factor output in the activated fibroblast [143]. A parallel mechanism couples the epigenome to the proximal oxidative effector of Section 3.3: fibroblast MeCP2 represses methylated SOD2, and it is the resulting loss of SOD2 that raises mitochondrial reactive oxygen species and drives cytoskeletal remodelling and ferroptosis, which MeCP2 knockdown reverses [144]. Environmental input is demonstrable at the AGT locus itself, a high-salt diet altering methylation and increasing human AGT expression in mice carrying the pro-hypertensive Hap-I human haplotype [145]. The therapeutic corollary is that DNA methyltransferases, histone acetyltransferases and histone deacetylases are druggable nodes controlling fibroblast activation state [146].

### 5.5. Non-Coding RNA Networks

MicroRNAs. miR-21 is the prototypical profibrotic species: enriched in cardiac fibroblasts, increased in failing myocardium, and acting through SPRY1 to derepress ERK–MAP kinase signalling, with silencing attenuating interstitial fibrosis and dysfunction [147]. Ang II drives its transcription through an osteopontin-dependent AP-1 mechanism [82], placing miR-21 squarely downstream of the axis under review, and silencing is antifibrotic in other cardiac models and in experimental HFpEF [148,149]. The miR-29 family acts in the opposite direction, targeting collagen and matrix transcripts directly; it falls in the peri-infarct region, where its loss permits matrix derepression [150]. That symmetry is deceptive, because cardiomyocyte-derived miR-29 promotes remodelling through Wnt signalling [151]: the same species is profibrotic or antifibrotic according to the cell that releases it, which is the central obstacle to systemic delivery. Of the remaining candidates, antimiR-132 has reached early clinical evaluation in heart failure [152] and miR-34 inhibition improves remodelling experimentally [153].

Long non-coding and circular RNAs, and vesicular transfer. Wisper is fibroblast-enriched, controls cardiac fibrosis and remodelling, and tracks fibrosis severity in human aortic stenosis tissue [154], whereas the mitochondrially encoded LIPCAR circulates, is dysregulated after myocardial infarction and predicts heart failure and mortality—a mechanistic candidate and a biomarker at once [155,156]. Circular RNAs are the least annotated class in the human heart and add a further layer to Wnt-dependent fibroblast activation [77]: circITGa9, found among more than 32,000 previously unannotated species in hypertrophic myocardium, aggravates fibrosis after transverse aortic constriction by binding tropomyosin 3, and blocking that interaction improves function and reduces fibrosis [157]. Transfer between cells is itself a modality rather than a disposal route: fibroblast-derived exosomes deliver microRNAs to cardiomyocytes and drive hypertrophy [158,159].

### 5.6. Metabolomic Signatures and Metabolic Reprogramming

Matrix synthesis is metabolically expensive. Collagen is approximately one-third glycine and rich in proline and hydroxyproline, and its production imposes specific biosynthetic demands that activated fibroblasts meet by reprogramming central carbon metabolism.

Fibroblast-intrinsic reprogramming. Myofibroblast differentiation is accompanied by a shift toward aerobic glycolysis with increased glutaminolysis, a pattern documented in renal and systemic sclerosis fibroblasts and increasingly in cardiac cells [160]. TGF-β activates mTORC1–ATF4 signalling to upregulate the de novo serine–glycine synthesis pathway, directly supplying the glycine required for collagen production; interruption of this axis limits collagen synthesis [161]. Mitochondrial function in the myofibroblast is itself a regulatory node governing the activated phenotype [162]. These findings identify fibroblast metabolism as a target class distinct from receptor and transcription factor inhibition.

Circulating metabolite signatures. Untargeted metabolomics in prospective cohorts links serum metabolites to incident heart failure [163,164], and several of those signatures are now mechanistic rather than merely correlative: branched-chain amino acid catabolic insufficiency promotes heart failure, with lymphatic-dependent BCAA accumulation contributing to experimental HFpEF [165,166]; acylcarnitine profiles reflecting incomplete fatty acid oxidation associate with diabetic heart failure [167]; and trimethylamine N-oxide, a gut microbiota-dependent metabolite, is associated with outcome in heart failure and has been proposed as a modifiable target [168].

Three mechanistic studies published in the last two years convert this correlative metabolomics into causal biology, and each identifies a node where metabolism and gene regulation meet. ATP citrate lyase, the enzyme generating acetyl-CoA for de novo lipogenesis, was upregulated in fibrotic areas of hearts infused with Ang II and phenylephrine; its silencing reduced fibrosis, and 13C tracing with chromatin immunoprecipitation in human cardiac fibroblasts linked lipogenic flux to histone acetylation at fibrogenic loci [169]. YAP activation in cardiac fibroblasts induced glycolysis and drove fibroinflammation while disrupting fibroblast identity, with chamber-specific differences in YAP and glycolytic activity detectable in human single-nucleus data [170]. In the cardiomyocyte compartment, the RNA-binding protein GRSF1 maintains branched-chain amino acid homeostasis post-transcriptionally, and its loss contributed to heart failure pathogenesis—providing a mechanism for the BCAA signatures repeatedly reported in human cohorts [171]. Metabolic reprogramming is therefore not a downstream consequence of fibroblast activation but one of its regulatory substrates.

Proteomic integration. Plasma proteomic signatures predict mortality in heart failure, associate with diastolic dysfunction and relate exercise blood-pressure responses to incident hypertension [172,173,174]. The programme of the multi-omics era is to integrate these layers rather than to rank them [88], with machine learning used to fuse them into clinically actionable predictions [175]. Table 2 sets out what each layer contributes, the evidence available in human myocardium and the principal limitation of each.

## 6. Emerging Biomarkers for Early Detection and Monitoring

### 6.1. Evaluation Framework

A biomarker of cardiac fibrosis must satisfy requirements that are more demanding than those for a biomarker of cardiac stress. It should reflect fibrotic activity rather than fibrotic burden alone if it is to guide early intervention; it should be tissue-attributable, since collagen turnover occurs in every organ; it should be analytically stable and standardised; and it should add information beyond natriuretic peptides, high-sensitivity troponin and CMR, which are already available. Natriuretic peptides remain the reference comparator for diagnosis and rule-out in suspected heart failure and are embedded in guideline algorithms [176], but they report wall stress rather than matrix biology, and it is precisely this orthogonality that motivates fibrosis-specific markers.

### 6.2. Collagen Turnover Peptides

Procollagen type I carboxy-terminal propeptide (PICP) and procollagen type III amino-terminal propeptide (PIIINP) are released stoichiometrically during fibril assembly and therefore index synthesis; carboxy-terminal telopeptide of collagen type I (CITP) indexes degradation. These are the oldest and, in several respects, the best-validated fibrosis biomarkers. Serum markers were shown three decades ago to track biochemically assessed myocardial fibrosis in hypertensive patients [177], and PICP was subsequently used to demonstrate the cardioreparative capacity of antihypertensive treatment [178]. Two landmark studies established that these analytes are treatment-responsive: losartan-dependent regression of biopsy-verified myocardial fibrosis was accompanied by reduced left ventricular chamber stiffness [179], and losartan and atenolol produced divergent effects on ultrasonic and biochemical fibrosis markers despite comparable blood pressure reduction [180]. This dissociation between pressure lowering and matrix response is among the strongest clinical arguments that Ang II acts on the matrix through pressure-independent mechanisms.

Collagen turnover markers also carry prognostic information, associated with outcomes in heart failure [181]. Their principal limitation is extracardiac origin: bone, skin, lung and liver contribute substantially to circulating concentrations, and renal clearance influences levels. The CITP:MMP-1 ratio has been proposed as a means of inferring crosslinking status rather than turnover volume, and integration with imaging improves interpretability [83]. How weakly these analytes track imaging burden is quantified by the collagen-marker substudy of the pirfenidone trial in heart failure with preserved ejection fraction: baseline ECV was unrelated to baseline PICP, CITP or their ratio; pirfenidone left PICP unchanged while reducing CITP and raising the PICP:CITP ratio at 13 and 26 weeks without a sustained effect at 52 weeks; and after adjustment, there was a weak relationship between change in ECV and change in log PICP (R^2^ = 0.16, *p* = 0.01) and log CITP (R^2^ = 0.12, *p* = 0.04)—16% and 12% of the variance, respectively—and none with the log PICP:CITP ratio [182]. Collagen turnover peptides and ECV are therefore not interchangeable readouts of the same process, and a trial powered on one should not be interpreted through the other. That dissociation is not the same as inertness: in the PARADIGM-HF matrix substudy, PINP and PIIINP fell further under sacubitril/valsartan than under enalapril in 1776 randomised patients, while baseline PIIINP predicted the primary outcome and change in sST2 and TIMP-1 tracked change in outcome [183]. The peptides are therefore treatment-responsive and prognostically live while remaining poor surrogates for imaging burden, which locates the problem in what they measure rather than in whether they measure anything. Part of that dissociation is likely analytical. These assays are directed at different cleavage products of the same molecule—PICP at the carboxy-terminal and PINP at the amino-terminal propeptide of procollagen I, the latter being the analyte used in the sacubitril/valsartan substudy above [183]—and the absolute concentrations that have proved informative were derived and validated within single cohorts, as with the PICP threshold of 108.1 ng/mL that stratified reverse remodelling and outcome in 1034 patients [184]. Within-study change is therefore more interpretable than absolute concentration, and published thresholds should be treated as cohort-specific until the assays are cross-calibrated [83].

### 6.3. Matricellular and Immune Mediators

Galectin-3 is macrophage-derived and mechanistically profibrotic [79]; as a clinical biomarker it is associated with outcome in heart failure, and its analytical characteristics and interpretive caveats have been reviewed [185]. Its major limitation is confounding by renal function and by systemic inflammation and fibrosis elsewhere [185].

Soluble ST2, the decoy receptor for IL-33, reflects the biomechanically induced IL-33/ST2 axis [186]. Meta-analysis supports independent prognostic value in chronic heart failure [187], and sST2 has the practical advantage of relative insensitivity to age, body mass index and renal function compared with galectin-3. It is, however, a marker of myocardial stress and inflammation rather than of collagen deposition specifically.

GDF-15 is a stress-responsive TGF-β superfamily member with consistent prognostic value across chronic heart failure cohorts and age strata [188,189], but it is among the least specific markers in this set, rising in inflammation, renal impairment, malignancy and ageing.

### 6.4. Circulating Non-Coding RNAs and Cell-Free DNA

Circulating microRNAs are attractive because they are stable in plasma, quantifiable by PCR, and potentially cell-type-informative; their diagnostic and prognostic evaluation in cardiovascular disease has been reviewed extensively [190]. Fibrosis-relevant candidates include miR-21 and miR-29 [147,150]. Practical translation is constrained by the absence of consensus normalisation strategies, by haemolysis artefacts, and by platform-dependent quantification, which together account for much of the inconsistency across published cohorts.

LIPCAR is the most clinically advanced circulating lncRNA in this domain, with dysregulation after myocardial infarction predicting subsequent heart failure development and mortality [155]; the wider non-coding RNA biomarker landscape has been surveyed recently [156]. Extracellular vesicle cargo offers a route to greater tissue attribution: exosome populations are routinely identified by their surface proteins [159], so capture directed at cell-of-origin markers rather than pan-exosomal tetraspanins is a plausible, though as yet unvalidated, way to enrich myocardial signal before RNA quantification.

Cell-free DNA methylation deconvolution is conceptually the most promising approach to the tissue-attribution problem. Because methylation patterns are cell-type-specific and are retained in the fragments released by dying cells, plasma cfDNA can in principle report both the identity and the quantity of the cells contributing to turnover [191]. A cardiac-specific application has recently been described, in which a methylated gene marker serves as a heart-specific cell-free DNA biomarker [192]. If a fibroblast-specific methylation signature can be defined and deconvoluted with adequate sensitivity, this would address the central specificity limitation of every analyte discussed above. The approach is early, and its sensitivity at the low fractional contributions expected from myocardial fibroblasts remains to be established; the broader role of cell-free nucleic acids in cardiovascular disease, as biomarkers and as mechanistic mediators, has recently been reviewed [193].

### 6.5. Imaging as a Comparator and as a Composite Partner

Any circulating biomarker must be judged against CMR, which currently defines the reference phenotype. Native T1 mapping and extracellular volume fraction (ECV) quantify diffuse interstitial fibrosis, are validated against biopsy-derived collagen volume fraction in dilated cardiomyopathy [194] (collagen volume fraction available in 24 patients), and carry prognostic weight: meta-analysis of 5384 patients demonstrates that both measures are higher in those who experience adverse outcomes and that ECV predicts events consistently across the heart failure spectrum (hazard ratio 1.20 per 1% increase, 95% CI 1.13–1.28), whereas native T1 is less predictive than ECV, particularly in heart failure with preserved ejection fraction [195]; independent prognostic value in dilated cardiomyopathy is also established [196]. ECV has been proposed specifically as a surrogate endpoint to enable antifibrotic drug development [197], explicitly as a phase 2 endpoint, with phase 3 trials still required to show event reduction. Late gadolinium enhancement, which detects focal replacement fibrosis, is prognostic in non-ischaemic dilated cardiomyopathy and across cardiomyopathies [198], but is insensitive to the diffuse interstitial expansion characteristic of Ang II-driven remodelling.

A conceptually distinct advance is molecular imaging of fibroblast activation. 68Ga-FAPI positron emission tomography visualises activated fibroblasts rather than deposited matrix [199], and comparison with histopathology in dilated cardiomyopathy supports its validity as an activity readout [200]. The evidence base has since broadened in three directions that matter for biomarker calibration. First, phenotypic discrimination: in a prospective case–control study of 81 participants, no myocardial [68Ga]FAPI-46 signal was detectable in healthy volunteers, whereas every patient with heart failure showed increased uptake—intense and infarct-localised in ischaemic cardiomyopathy (mean SUVmax 3.2) and diffuse, lower-intensity and basal-septal in non-ischaemic disease (2.3), the latter irrespective of late gadolinium enhancement; higher baseline uptake was associated with less improvement in ejection fraction on optimal medical therapy [201]. Second, temporal resolution: after acute myocardial infarction, fibroblast activation involved a greater myocardial fraction than late gadolinium enhancement (27.8% ± 12.4% versus 15.2% ± 10.6%), peaked at one to two weeks, declined over twelve weeks yet remained detectable in infarcts years old, and predicted subsequent left ventricular dilatation and systolic dysfunction [202].

Third, reversibility: in mice infused with Ang II and phenylephrine, longitudinal FAPI-PET tracked the transition of FAP-positive fibroblasts toward a FAP-negative phenotype after the infusion was withdrawn, establishing the signal as a dynamic rather than cumulative measure [203]. This distinction—activity versus burden—is exactly the one that circulating biomarkers also need to make, and FAPI-PET now offers a reference standard with a characterised time course and a demonstrated response to withdrawal of the fibrogenic stimulus, against which candidate “activity” biomarkers can be calibrated. Molecular imaging is also beginning to address matrix composition rather than matrix volume: a peptide-based probe selective for collagen type III has been validated for in vivo molecular CMR in murine myocardial infarction, mapping spatiotemporal changes in COL3 turnover that ECV cannot resolve and tracking response to treatment [204]. If this translates, the collagen I/III balance that circulating peptides can only infer becomes directly measurable.

Three constraints qualify this reference-standard role: generator-dependent tracer supply, absence of any cardiac reimbursement indication, and a procedure standard written for oncology that requires cardiac adaptation before quantitative values can be pooled [205]; FAPI-PET is therefore a reference standard within centres rather than across them. The leading candidate biomarkers, their analytical maturity and the evidence gap that currently blocks each from clinical use are compared in Table 3.

### 6.6. The Case for Multimarker and Multimodal Strategies

No single analyte satisfies all four criteria in Section 6.1, and it is unlikely that one will. The defensible strategy is composite: a synthesis marker (PICP) to report fibrogenic activity, a mediator marker (galectin-3 or sST2) to report the driving pathway, and an imaging measure (ECV) to report accumulated burden, interpreted jointly and, ideally, conditioned on genotype. Precision-medicine frameworks for cardiovascular biomarkers increasingly adopt this architecture [175,206], and contemporary appraisals of NT-proBNP, sST2 and galectin-3 in heart failure management—and of biomarkers across the acute heart-failure-care continuum—reach the same conclusion from the clinical side: each analyte adds prognostic information, none isolates matrix biology [207,208].

Two developments indicate how the specificity ceiling might be raised without waiting for a single fibrosis-specific molecule. High-throughput affinity proteomics can define multi-protein signatures of the fibrotic phenotype: a plasma signature of 39 proteins and one 42-protein cluster associated with elevated CMR-derived ECV independently of conventional risk factors, and 34 of those 39 proteins predicted incident heart failure over a mean of 9.8 years in an independent cohort of 3223 participants, with enrichment for T-cell activation, tumour necrosis factor signalling and tissue repair programmes [209]. Separately, models predicting ECV from routinely available clinical variables—without any tomographic imaging—achieved optimism-corrected C-statistics of 0.78 in derivation (*n* = 1663) and 0.74 in validation (*n* = 1578) for ECV ≥ 27%, and predicted ECV itself associated with adverse outcome (hazard ratio 2.21, 95% CI 1.84–2.66) [210]. Neither approach is matrix-specific in the molecular sense, but both produce a deployable fibrotic phenotype from measurements that already exist, and both are testable as enrichment tools now. Two methodological requirements are non-negotiable before such panels can be evaluated: prespecified analytical standardisation, and demonstration of incremental discrimination and reclassification over natriuretic peptides and imaging, rather than association alone.

## 7. Clinical and Translational Perspectives

### 7.1. What Established RAAS Blockade Does—And Does Not—Achieve

**Figure 4:**
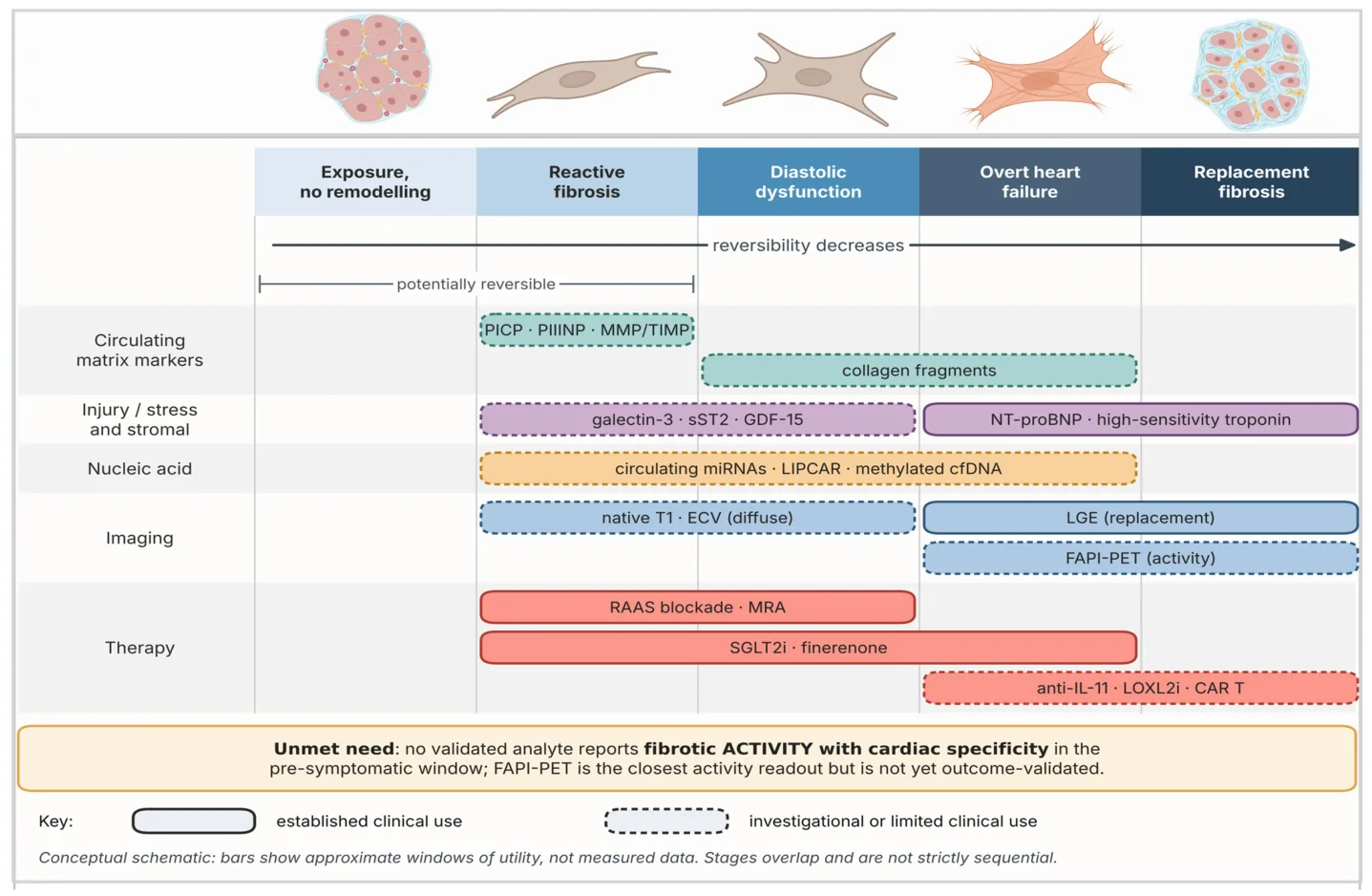
Detection and intervention opportunities shift as fibrotic remodelling advances. The upper band shows the corresponding tissue state at each stage, from the quiescent spindle-shaped fibroblast through the activated myofibroblast to a matrix-dominated scar in which the activated cell population recedes. Horizontal bars indicate the disease window in which each biomarker class, imaging modality or therapeutic strategy currently provides information or benefit, plotted against an ordinal severity axis along which reversibility decreases. That axis is ordinal by construction: the loss of reversibility is inferred from the regression of extracellular volume fraction and collagen volume fraction reported after afterload relief or RAAS blockade in the studies discussed in Section 6 and Section 7, and has not been measured as a continuous function of fibrotic burden. Collagen turnover peptides and nucleic-acid markers are informative earliest; natriuretic peptides and late gadolinium enhancement report established disease. Solid bars denote established clinical use and dashed bars investigational or limited clinical use. The principal unmet need, stated beneath the panel, is a cardiac-specific readout of fibrotic activity in the pre-symptomatic window. The schematic indicates approximate windows of utility, not measured data.

The disease windows in which the biomarkers, imaging modalities and therapies discussed in this section are informative or effective are mapped in Figure 4. ACE inhibitors, ARBs and MRAs reduce morbidity and mortality in heart failure, and part of that benefit is plausibly antifibrotic. The losartan studies described in Section 6.2 provide direct human evidence that Ang II blockade can regress biopsy-verified myocardial fibrosis and reduce chamber stiffness, and that this effect is not fully explained by blood pressure reduction [179,180]. A systematic review of studies relating ACE inhibition to serum PIIINP and PICP after myocardial injury supports a class antifibrotic effect at the level of collagen turnover markers, while noting that no recommendation currently exists for prescribing these agents on a fibrosis-prevention indication [211].

Three limitations qualify this achievement. First, incomplete pathway suppression: chymase-dependent Ang II generation, if it operates in vivo (its contribution is contested [13,14,15]), could proceed despite ACE inhibition, and aldosterone breakthrough restores mineralocorticoid signalling during chronic therapy. Second, heterogeneity of response, which is the clinical manifestation of the genomic and molecular variance catalogued in Section 5. Third, timing: by the time fibrosis is detectable by current clinical standards, crosslinked matrix and terminally differentiated fibroblast states may substantially limit reversibility (Section 3.5, Section 4.2 and Section 4.4).

Angiotensin receptor–neprilysin inhibition has been examined specifically for antifibrotic effect. Analyses of sacubitril/valsartan have addressed whether the benefit includes a matrix component [212,213], and the extracellular matrix biomarker substudy of PARADIGM-HF supplies the randomised evidence: in 1776 patients with paired samples, aldosterone, sST2, TIMP-1, MMP-9, PINP and PIIINP fell further under sacubitril/valsartan than under enalapril at eight months, and baseline sST2, TIMP-1 and PIIINP predicted the primary outcome, with change in sST2 and TIMP-1 tracking change in outcome [183]; a smaller, uncontrolled series of 26 patients report concordant movement [214]; a randomised CMR study has evaluated reverse remodelling of left ventricular hypertrophy [215]. Finerenone, a non-steroidal MRA with a distinct tissue distribution and safety profile relative to steroidal agents, reduced cardiovascular events in chronic kidney disease with type 2 diabetes [216,217] and reduced the composite of total worsening heart failure events and cardiovascular death, driven by the heart-failure component in heart failure with mildly reduced or preserved ejection fraction [218]—the population in which interstitial fibrosis is most pathophysiologically central. The indication has since been extended beyond diabetes: in 1584 adults with chronic kidney disease and no diabetes, finerenone slowed estimated glomerular filtration rate (eGFR) decline over 32 months (mean annual change −3.3 versus −4.0 mL/min/1.73 m^2^; difference 0.7, 95% CI 0.3–1.1; *p* < 0.001) and reduced a composite kidney or cardiovascular outcome (hazard ratio 0.77, 95% CI 0.60–0.99), with more hyperkalaemia (17.0% versus 13.3%) [219]; the pharmacological and clinical questions that still separate non-steroidal from steroidal agents have been appraised in parallel [220]. Direct imaging evidence that mineralocorticoid receptor blockade modifies the human interstitium also now exists, though it is modest: in a randomised trial in non-obstructive hypertrophic cardiomyopathy, native T1 fell within the eplerenone arm (1315 ± 134 ms to 1259 ± 92 ms at 12 months; *p* = 0.041) with no change under placebo, but the between-group difference did not reach significance (*p* = 0.07) and functional and diastolic measures were unchanged [221]. SGLT2 inhibitors, whose heart failure benefit is not fully explained by glycaemic or haemodynamic effects, attenuate oxidative stress and fibrosis experimentally [222,223,224], though a direct antifibrotic mechanism in humans remains to be demonstrated. The closest human evidence is a longitudinal controlled study in diabetic heart failure with preserved ejection fraction, in which dapagliflozin produced a greater reduction in CMR-derived ECV than placebo (−3.5%, 95% CI −4.2 to −2.8, versus −0.8%, −1.3 to −0.4; *p* < 0.001) alongside greater reduction in left ventricular mass index [225]; the cohort is small and the finding requires replication with blinded endpoint adjudication before a disease-modifying interstitial effect can be claimed for the class.

### 7.2. Emerging Therapeutic Targets

Fibroblast-directed cell therapy. The most conceptually significant recent advance is the demonstration that chimeric antigen receptor T cells targeting fibroblast activation protein can eliminate activated cardiac fibroblasts and reverse established fibrosis in mice [226]. The subsequent development of transiently expressed CAR T cells generated in vivo by targeted lipid nanoparticle delivery of modified mRNA addresses the central safety objection—persistence of cytotoxic cells directed against a marker also expressed in wound healing—by restricting the effector window [227]. FAP-directed CAR natural killer cells represent a further variation on this approach [228]. The unresolved questions are the specificity of FAP for pathological versus reparative fibroblasts and the consequences of ablating a population required for scar integrity.

Crosslinking inhibition. LOXL2 inhibition targets the step that converts reversible matrix expansion into durable stiffening [85], and a small-molecule LOXL2 inhibitor with demonstrated target engagement has advanced in preclinical evaluation [86]. The clinical case is strengthened by the observation that plasma LOXL2 is elevated in heart failure secondary to hypertension or aortic stenosis and correlates with left ventricular mass index, and by phase 1 data for the small-molecule inhibitor SNT-5382 showing a pharmacokinetic profile capable of high and prolonged LOXL2 inhibition on once-daily oral dosing after preclinical reduction of fibrosis in a myocardial infarction model [86]. This class is attractive because it does not require suppression of fibroblast activation itself.

Cytokine-directed therapy. IL-11 blockade is mechanistically appealing because it acts downstream of TGF-β within the fibroblast, potentially preserving the homeostatic functions of TGF-β while abolishing the fibrogenic output [45]. That appeal has to be weighed against the clinical record of targets downstream of TGF-β: pamrevlumab, built on the comparable rationale of blocking the CCN2 amplifier, failed in phase 3 in idiopathic pulmonary fibrosis [229]. Mechanistic proximity to the fibrogenic output has not so far translated into a therapeutic index, and IL-11 blockade inherits that burden of proof. Delivery, however, may be rate-limiting: a single intrapericardial injection of an IL-11-blocking antibody produced only modest attenuation of post-infarction fibrosis in mice, whereas encapsulation in a reactive oxygen species-responsive hydrogel prolonged retention at the injury site and markedly improved efficacy, reducing scar fibrosis and tissue stiffness and improving left ventricular contractility [230].

Epigenetic therapy. BET inhibition attenuates fibroblast activation and macrophage polarisation [139,141], and apabetalone has cardiovascular outcome data [142], but pan-BET inhibition lacks cell selectivity and its long-term safety in a chronic indication is unresolved.

Direct cardiac reprogramming. Conversion of resident fibroblasts into induced cardiomyocytes shifts profibrotic fibroblasts to a quiescent, antifibrotic state and converts a small fraction—about 2% of resident fibroblasts—into induced cardiomyocytes, reducing fibrosis and improving contractile function in chronic experimental infarction [231].

Other targets. Modified citrus pectin and related galectin-3 inhibitors reduce experimental fibrosis [232]; pirfenidone reduced myocardial extracellular volume in a randomised phase 2 trial in heart failure with preserved ejection fraction [233] (double-blind and placebo-controlled, with a between-group difference in ECV of −1.21%, 95% CI −2.12 to −0.31, among 94 patients randomised and 80 analysed), with accompanying analysis of collagen turnover markers [182]; relaxin-based approaches retain preclinical support despite the neutral phase 3 experience in acute heart failure [234]. Direct ALK5 inhibition remains constrained by the cardiac valvular and other toxicities observed with broad TGF-β pathway blockade, which is the principal reason the field has moved toward downstream and cell-selective nodes.

### 7.3. Precision Phenotyping: An Integrated Clinical Framework

The practical synthesis of this review is that patients at risk of Ang II-driven fibrotic remodelling could be stratified along three orthogonal axes: genomic susceptibility (ancestry-appropriate PRS supplemented by rare variant screening where indicated, and RAAS pharmacogenomic genotype), biochemical activity (a composite of collagen synthesis and mediator markers), and structural burden (CMR ECV, with FAPI-PET where available to distinguish active from quiescent fibrosis). High activity with low burden is hypothesised to identify the group in whom early intervention would be most effective and in whom antifibrotic trials would be most efficiently enriched; this prediction has not been tested; low activity with high burden identifies established, probably crosslinked fibrosis for which the therapeutic objective differs. The phenotype in which this stratification is most needed is HFpEF, where roughly half of patients show left ventricular hypertrophy or concentric remodelling; fibrotic remodelling contributes to diastolic stiffness and is associated with poor prognosis, and no evidence-based therapy is mechanistically directed at fibrosis or hypertrophy alone [235].

### 7.4. Pharmacogenomics of RAAS Blockade

Genotype-guided RAAS therapy remains an aspiration rather than a practice, and the reason is the quality of the evidence rather than any shortage of candidate variants. The most-cited positive result is a 31-patient, candesartan-only proof-of-concept substudy in which AGTR1 A1166C genotype tracked the two-week blood-pressure response and the 24-week NT-proBNP change; it tested ten polymorphisms without a placebo comparator or correction for multiple testing, enrolled only White participants of whom 93.5% were men, and found no association for the remaining variants, including ACE I/D, AGT Met235Thr/Thr174Met and CYP11B2 −344C/T [236]. The authors themselves call for validation before clinical use. Across the wider literature the pattern is the same: small exploratory studies, no prospective randomised evidence of genotype-guided benefit, and derivation overwhelmingly in European-, East Asian- and African American-ancestry populations, with Hispanic/Latino participants appearing as subgroups within United States trials rather than as study populations in their own right [237]. The Mexican studies that do exist as populations in their own right have addressed susceptibility to hypertension and coronary artery disease, not response to treatment [90,91,98].

For admixed Latin American populations, local pharmacogenomic data are therefore a prerequisite rather than an optional refinement. The minimum viable first step is not a new genome-wide effort but systematic genotyping of the small set of variants with mechanistic or association support—ACE I/D, whose pooled association with heart failure risk is null [89] and which is for that reason worth prospective testing, AGT rs4762 and rs5050, AGTR1 A1166C and CYP11B2 −344C/T—within cohorts that already carry deep cardiovascular phenotyping, so that modest sample sizes remain usable against a single harmonised remodelling endpoint. Part of that groundwork exists: ACE and AGTR1 variants have been genotyped in Mexican hypertensive cohorts with serum ACE measured by haplotype [90,98], so what is missing is not genotyping capacity but the prospective link to treatment response and to a fibrosis readout, and A1166C itself, which those cohorts did not include. Admixture then has to be modelled rather than adjusted for: global ancestry proportions are insufficient, whereas local-ancestry inference assigns ancestry segment by segment [238], and it matters here because the variants of interest differ in frequency across the Indigenous American, European and African components of the genome (Table 4).

The gradient is steep for AGTR1 1166C and CYP11B2 −344C and negligible for the two AGT variants, and the ACE insertion/deletion is absent from the resource altogether, a 287-base-pair Alu indel being poorly ascertained by short-read sequencing—itself a reason to genotype it directly rather than impute it from a reference panel. The 9p21 coronary locus is the paradigm case of such ancestry-dependent architecture, its two common risk haplotypes stratifying risk in every population studied except those of African origin, in which they are virtually absent [239]. Polygenic instruments must likewise be evaluated rather than imported, for the reasons set out in Section 5.2 and because published coronary scores disagree materially with one another when applied to the same individuals [240]; multi-population scores derived and validated across White, Black and Hispanic participants [239] and the published implementation pipeline, including calibration of score mean and variance by genetic ancestry [241], together provide the template for assessing a candidate score in a new population. The realistic deliverable is not a Latin American polygenic score for cardiac fibrosis, which current sample sizes cannot support, but a calibrated estimate of how much of the reported RAAS pharmacogenomic signal survives in an admixed population. The arithmetic constrains the design: detecting a per-allele effect of 0.2 standard deviations at a minor allele frequency of 0.30 requires about 470 participants at a nominal 5% level and about 2400 at genome-wide significance, and a genotype-by-treatment interaction requires roughly four times the main-effect sample—numbers attainable only through pooled regional cohorts or multi-ancestry consortia with Latin American strata.

**Table 4:** Allele frequencies of the candidate RAAS variants across gnomAD v4 ancestry groups, showing the ancestry-dependent architecture that local-ancestry inference is required to resolve. The admixed American group is a United States-based proxy rather than a Latin American sample, which is itself part of the argument for region-specific genotyping. The ACE insertion/deletion has no entry in the resource. Data from [242].

Variant	African/African American	Admixed American	Non-Finnish European
AGTR1 1166C	5.9%	28.6%	30.1%
CYP11B2 −344C	22.9%	43.1%	45.2%
AGT 174Met	6.3%	11.9%	12.2%
AGT −20C	14.7%	18.6%	16.9%
ACE I/D	not ascertained	not ascertained	not ascertained

### 7.5. Trial Design Implications

Three design consequences follow. First, enrichment: trials should select patients by fibrotic burden and, once an activity readout is outcome-validated, by fibrotic activity, not by ejection fraction alone. Second, endpoints: ECV change is a biologically plausible candidate surrogate with an explicit rationale for antifibrotic development [197], whose own scheme requires phase 2 trials showing fibrosis regression before phase 3 trials showing event reduction; baseline ECV is prognostic [210], but it has not been shown that a drug-induced change in ECV predicts clinical benefit, and PIROUETTE provides a template in which a fibrosis-directed agent was evaluated against an imaging endpoint in HFpEF [233], having selected patients by that same ECV ≥ 27% threshold, although it was not designed or powered to test clinical outcomes. Third, stratification: prespecified genomic and biomarker strata should be built into the analysis plan, since a treatment effect confined to a molecularly defined subgroup may be diluted in an unstratified population; strata therefore need prespecified interaction tests and adequate power.

Enrichment need not require universal imaging. Applied at a probability threshold of 0.69 and 80% specificity, imaging-free predicted ECV would have obviated 3940 CMR scans in a hypothetical phase 3 trial with an ECV ≥ 27% entry criterion, at the cost of screening 3052 additional participants and enrolling 705 more [210] (C-statistic 0.78 in derivation and 0.74 in external validation; at the 0.69 threshold, sensitivity for ECV ≥ 27% was 53% and positive predictive value 72%, in two single-centre cohorts from Manchester and Pittsburgh, 86% White)—a trade whose cost-saving the authors anticipate but did not quantify, and it is untested in Latin American centres (where it is undocumented whether scanner access or screening capacity is the binding constraint) or in non-White populations. A trial that has completed recruitment illustrates an enriched, fibrosis-endpoint design: Reduce-MFA DZHK25 randomises patients with severe aortic stenosis and high baseline fibrotic burden (CMR ECV ≥ 25.9%) undergoing transcatheter aortic valve implantation to standard of care alone, standard of care plus spironolactone, or standard of care plus spironolactone and low-dose dihydralazine, with reduction in the ECV-derived left ventricular matrix volume at 12 months as the primary endpoint—analysed as a hierarchical composite in which all-cause mortality is the first step—and with reverse remodelling, symptoms and clinical events as secondary objectives [243] (PROBE design: open-label treatment with blinded outcome assessment; dihydralazine is dosed by acetylator status confirmed by genetic testing, which makes it the rare cardiac antifibrotic trial with a pharmacogenetically guided element already built in). Its entry criterion is a fibrosis threshold, its endpoint is a fibrosis measure, and its intervention is repurposed—the three features that make an antifibrotic trial feasible before a fibroblast-selective agent reaches late-phase development. The cost of that entry criterion is visible in its recruitment: 384 patients enrolled at 13 centres between January 2022 and August 2024 yielded 153 randomised [243]. An ECV threshold of 25.9% also bounds inference to a high-burden stratum, which does not transfer to lesser interstitial expansion, to non-valvular aetiologies or to HFpEF; prespecifying ECV as a continuous covariate with a treatment-by-ECV interaction keeps the threshold testable rather than assumed.

### 7.6. Synthesis of the Evidence Base

The preceding sections draw on two evidence streams of unequal maturity, and separating them makes the current translational position explicit. Table 5 summarises the human studies that support the genomic, biomarker and therapeutic claims made above; the in vitro and in vivo model studies that establish the underlying mechanisms are tabulated in Supplementary Table S2. The asymmetry is informative: causal, cell-autonomous evidence for almost every node in the fibrotic cascade exists in rodent models, whereas the human evidence is predominantly associative—genetic association, imaging or biomarker correlation, and a small number of randomised trials in which a fibrosis measure was a secondary or mechanistic endpoint rather than the primary outcome. No completed trial has used fibrotic activity as both the entry criterion and the primary endpoint. This gap, rather than any deficiency of mechanistic understanding, is what currently limits antifibrotic translation, and it directly motivates the trial-design requirements set out in Section 7.5 and the limitations catalogued in Section 8.

**Table 5:** Human studies supporting the genomic, biomarker and therapeutic claims of this review, by evidence type. Abbreviations: BP, blood pressure; CKD, chronic kidney disease; CV, cardiovascular; DCM, dilated cardiomyopathy; DEG, differentially expressed gene; EF, ejection fraction; HR, hazard ratio; ICM, ischaemic cardiomyopathy; IHD, ischaemic heart disease; LGE, late gadolinium enhancement; LV, left ventricular; LVH, left ventricular hypertrophy; MI, myocardial infarction; MR, Mendelian randomisation; PK, pharmacokinetics; TAVI, transcatheter aortic valve implantation. Other abbreviations are defined in the abbreviation list.

Study Type/Design	Population (n Where Stated)	Exposure or Intervention	Fibrosis-Related Readout	Principal Finding	Ref.
Genetic association meta-analysis	Pooled hypertensive and HF cohorts	*ACE* I/D genotype	HF risk	No significant association between ACE I/D genotype and HF risk under any of four genetic models (17 studies, 5576 participants); ACE activity not measured	[89]
Genetic association meta-analyses	Hypertension and IHD cohorts	*AGT* M235T/promoter haplotype	Plasma angiotensinogen; BP; IHD	Association with higher plasma angiotensinogen and with hypertension (pooled OR 1.08 for MT and 1.19 for TT in White subjects); no association with ischaemic heart disease or myocardial infarction in either ethnic group	[92,93]
Genetic association studies and meta-analysis of LV structure	Hypertensive cohorts	*CYP11B2* −344C/T	LV hypertrophy and LV structural indices	Associated with LVH and LV structural indices	[95,96]
Prospective genetic cohort	755 Chinese patients with essential hypertension, 11-year follow-up	*CYP11B2 −344C/T*	Major adverse cardiovascular events	−344CC genotype independently predicted MACE (HR 2.05), coronary artery disease (HR 1.75) and stroke (HR 2.59), but not CAD-related death	[97]
Multi-ancestry GWAS with mediation analysis	Multi-ancestry population cohorts	Circulating angiotensinogen	Angiotensinogen concentration; BP; hypertension	115 variants; lead signal differs by ancestry (rs4762, rs5050, rs16852311); BP effect mediated by angiotensinogen	[99]
GWAS with Mendelian randomisation	Resistant hypertension cases and controls	Genome-wide genotype	Serum potassium; hyperaldosteronism; drug response	24 variants; risk alleles track lower potassium; resistant hypertension largely a hyperaldosteronism phenotype	[100]
GWAS with MR and plasma proteomics	Large HF meta-cohorts	Genome-wide genotype and plasma proteome	HF risk; causal protein nomination	Prioritises druggable targets within HF loci rather than descriptive association	[102]
GWAS	Dilated cardiomyopathy cohorts	Genome-wide genotype	DCM risk; myocardial resilience	Identifies modifiers of the response to insult rather than the insult itself	[103]
Imaging-genetics GWAS	Large CMR imaging cohorts	Genome-wide genotype	CMR native T1 (interstitial fibrosis)	Loci associated with the fibrotic phenotype itself; most directly relevant genomic evidence to date	[104]
Single-nucleus RNA sequencing	20 LV samples: non-failing, DCM, ICM (incl. scar)	Disease state	Fibroblast state composition and trajectory	Resident perivascular/interstitial states depleted in HF; scar and interstitial programmes differ	[126]
Single-nucleus RNA sequencing	Pooled HFpEF myocardial biopsies; 48,886 nuclei	HFpEF versus control	Cell-type transcriptional perturbation	Fibroblasts most perturbed population (5905 DEGs versus 5159 in cardiomyocytes)	[128]
Spatial whole-transcriptome profiling	90 tissue cores from 44 participants	Disease state	Spatially resolved transcriptome	Anchors fibroblast states to histology; compartmentalisation accuracy limited for fibroblasts and endothelium	[129]
Integrated proteomic, transcriptomic and genetic analysis	Aortic stenosis cohorts with independent replication	Pressure overload	Interstitial fibrosis; clinical outcome	Prioritises circulating mediators of fibrosis, hypertrophy and oxidative stress associated with outcome	[130]
Observational biochemical studies	Hypertensive patients	Hypertension; antihypertensive treatment	Serum PICP vs. LV hypertrophy, diastolic function and arrhythmias in patients; vs. biopsy collagen and hydroxyproline in SHR rats	Serum collagen peptides track biochemically assessed fibrosis; treatment shows cardioreparative capacity	[177,178]
Prospective biomarker cohort	Post-MI patients	Myocardial infarction	Circulating LIPCAR (lncRNA)	Dysregulated after MI; predicts incident HF and mortality	[155]
Methylation biomarker study	Breast cancer patients receiving anthracyclines	Anthracycline exposure	Methylated *PIH1D1* in plasma	Heart-specific cell-free DNA methylation marker of cardiac remodelling	[192]
Biomarker and phase 1 pharmacology	HF from hypertension or aortic stenosis; healthy volunteers	LOXL2 inhibitor SNT-5382	Plasma LOXL2; LV mass index; PK	Plasma LOXL2 elevated and correlates with LV mass index; PK supports prolonged inhibition on once-daily dosing	[86]
Biopsy validation	74 patients with dilated cardiomyopathy (collagen volume fraction from endomyocardial biopsy in 24)	Dilated cardiomyopathy of varying severity	CMR ECV versus histological collagen volume fraction	ECV tracks histological fibrosis (r = 0.85)	[194]
Prognostic meta-analysis	19 studies, 5384 patients with heart failure	HF of mixed aetiology	CMR native T1 and ECV	ECV predicts events across the HF spectrum (HR 1.20 per 1% increase, 95% CI 1.13–1.28); native T1 less predictive than ECV, particularly in HFpEF	[195]
Prospective case–control imaging study	81 participants: healthy volunteers and HF	[^68^Ga]FAPI-46 PET	Myocardial FAP-directed uptake	No signal in healthy volunteers; all HF patients positive; diffuse basal-septal pattern in non-ischaemic disease independent of LGE	[201]
Longitudinal imaging cohort	Patients after acute MI	Myocardial infarction	FAPI-PET versus LGE	Activated fraction exceeds LGE (27.8% versus 15.2%); peaks at 1–2 weeks; predicts subsequent LV dilatation and dysfunction	[202]
Affinity proteomics with external validation	ECV-phenotyped cohort; validation *n* = 3223	Fibrotic phenotype	CMR ECV; incident HF over mean 9.8 years	39-protein plasma signature associated with elevated ECV; 34 proteins predict incident HF	[209]
Prediction model development and validation	Derivation *n* = 1663; validation *n* = 1578	Routine clinical variables	Predicted CMR ECV ≥ 27%	C-statistic 0.78/0.74; predicted ECV associated with outcome (HR 2.21, 95% CI 1.84–2.66)	[210]
Systematic review	Studies of patients after myocardial injury	ACE inhibition	Serum PIIINP and PICP	Supports a class effect on collagen turnover markers; no fibrosis-prevention indication currently exists	[211]
Randomised trials and biomarker/CMR substudies	HF populations	Sacubitril/valsartan	Fibrosis and inflammation markers; LV mass	Sacubitril/valsartan lowered serum markers of extracellular matrix turnover in PARADIGM-HF; reverse remodelling of LV hypertrophy evaluated by CMR	[183,212,214,215]
Randomised outcome trial	HF with mildly reduced or preserved EF	Finerenone (non-steroidal MRA)	Composite of worsening HF events and cardiovascular death (benefit driven by HF events)	Benefit in the population in which interstitial fibrosis is most central	[218]
Randomised outcome trial	1584 adults, CKD without diabetes	Finerenone	eGFR slope; composite kidney/CV outcome	Slower eGFR decline (difference 0.7 mL/min/1.73 m^2^/year) and HR 0.77 for the composite; hyperkalaemia 17.0% versus 13.3%	[219]
Randomised clinical trial	Non-obstructive hypertrophic cardiomyopathy	Eplerenone versus placebo	CMR native T1 at 12 months	Within-arm T1 fall (1315→1259 ms, *p* = 0.041); between-group difference not significant (*p* = 0.07)	[221]
Longitudinal controlled study	Diabetic HFpEF	Dapagliflozin versus placebo	CMR ECV; LV mass index	Greater ECV reduction (−3.5% versus −0.8%, *p* < 0.001); small cohort, requires replication	[225]
Randomised phase 2 trial with collagen-marker substudy	HFpEF	Pirfenidone	CMR ECV; PICP, CITP, PICP:CITP	ECV reduced; PICP unchanged, CITP reduced at 13 and 26 weeks but not sustained at 52 weeks; ΔECV–Δpeptide correlation weak (R^2^ = 0.12–0.16, i.e., 12–16% of variance)	[182,233]
Randomised trial, recruitment completed (384 enrolled, 153 randomised); Reduce-MFA DZHK25	Severe aortic stenosis with ECV ≥ 25.9% undergoing TAVI	Standard of care alone; + spironolactone; + spironolactone and low-dose dihydralazine	Reduction in ECV-derived LV matrix volume at 12 months (primary, as a hierarchical composite with all-cause mortality)	Prototype of molecular enrichment: fibrosis threshold for entry, fibrosis measure as endpoint	[243]

### 7.7. Multi-Omic Integration and Machine Learning

Translating layered molecular data into decisions requires models that combine them, and that work has begun. Unsupervised integration of proteomic, metabolomic and clinical data resolves heart failure with preserved ejection fraction into phenotypes with distinct trajectories [244], and machine-learning models applied to routinely acquired data can identify undiagnosed disease before it is clinically apparent [245]. Two caveats bound their current standing. Such models inherit the ancestry and referral biases of their training cohorts (Section 8), and none has yet been shown to alter management in a prospective trial, so their contribution at present is to generate hypotheses and to prioritise patients for confirmatory measurement rather than to direct treatment.

## 8. Limitations of the Current Evidence Base

Several constraints temper the conclusions drawn above and should be stated explicitly.

Endpoint heterogeneity. Studies grouped here as measuring cardiac fibrosis use readouts that are not interchangeable: histological collagen volume fraction, extracellular volume fraction or native T1 on CMR, late gadolinium enhancement as a binary presence, and circulating collagen propeptides each report a different aspect of matrix biology at a different spatial scale. Effect sizes are therefore not poolable across the studies cited here, and apparent disagreement between reports sometimes reflects endpoint choice rather than biology.

Model validity. Subcutaneous Ang II infusion produces rapid, severe hypertension and fibrosis over days to weeks, whereas human hypertensive heart disease evolves over decades. Separating pressure-dependent from pressure-independent effects requires deliberate experimental design, and in one comparative study in Ang II-infused mice, apparent blood-pressure independence of the hypertrophic effect rested on non-significant tail-cuff differences between small groups (*n* = 6–8), and fibrosis, modest at 1–2% of the left ventricular area, did not differ between treatments [246]. Murine collagen isoform composition, heart rate and ECM turnover kinetics differ from human, limiting quantitative extrapolation.

Species and sex. Sex differences in cardiac structural remodelling are consistent and substantial [247], with GPER-dependent mechanisms contributing under pressure overload [248]. In humans with aortic stenosis, women carry a lower late-gadolinium-enhancement burden than men at comparable extracellular volume, yet fibrosis predicts post-intervention mortality in both sexes without a significant interaction by sex [249]. Most mechanistic studies nonetheless use male animals exclusively, and most biomarker thresholds are not sex-specific.

Ancestry. The genomic literature reviewed in Section 5 is overwhelmingly European-ancestry in origin. Polygenic scores transport poorly across ancestries [108,109,110], and this is a limitation of the evidence base, not a caveat to be appended to its application.

Tissue availability. Human cardiac fibroblast biology is inferred largely from explanted end-stage hearts and from non-failing donor hearts declined for transplantation. The early, potentially reversible phase of Ang II-driven fibrosis is not directly observable in human tissue at scale.

Publication, terminology and retrieval. Negative results in antifibrotic pharmacology are systematically under-reported, and fibroblast activation-state nomenclature is not standardised across laboratories, which complicates cross-study synthesis of single-cell data. Retrieval was confined to Scopus and PubMed/MEDLINE; Embase and Web of Science were not interrogated, so material indexed only in those resources may not be represented.

Assumptions not tested here. This review does not attempt formal quantitative synthesis; effect sizes reported in the cited meta-analyses are not re-derived, and no assessment of between-study heterogeneity beyond that reported by the original authors was performed.

## 9. Conclusions and Future Directions

Chronically elevated angiotensin II drives cardiac fibrosis through a now well-defined architecture: AT1R engagement, amplified by local chymase-dependent peptide generation and by ligand-independent mechanical activation and opposed by the ACE2–Ang-(1-7)–Mas axis, converges through NADPH oxidase-derived oxidant signalling and sterile inflammation on resident Tcf21-lineage fibroblasts, which transition under TGF-β–SMAD2/3 and IL-11 control to a matrix-producing myofibroblast state locked in by a mechanotransductive stiffness loop. That response varies between individuals, and the variance is now resolvable across germline, epigenomic, single-cell and metabolic layers.

Biomarker development has not kept pace. Collagen turnover peptides remain the most biologically interpretable analytes and the only ones with demonstrated treatment responsiveness in human myocardial fibrosis, but they lack tissue specificity; galectin-3, sST2 and GDF-15 report pathway activity or generalised stress rather than matrix deposition. Cell-free DNA methylation deconvolution is the most promising route to tissue attribution and the least mature.

Six priorities follow.

1. Conduct genome-wide and multi-omic discovery for fibrosis endophenotypes in ancestrally diverse cohorts, including Latin American populations, using imaging-derived quantitative traits rather than clinical endpoints.
2. Define a cardiac fibroblast-specific methylation signature and establish whether it is detectable and quantifiable in plasma cell-free DNA at the fractional contributions expected in early remodelling.
3. Determine whether a methylation- or chromatin-locked activated fibroblast state exists in human myocardium, as it does in renal fibrosis, and whether it is pharmacologically reversible.
4. Establish standardised, prespecified analytical protocols for circulating non-coding RNA quantification, without which cross-cohort synthesis will remain uninterpretable.
5. Test cell-selective antifibrotic strategies—transient FAP-directed effectors, IL-11 blockade, LOXL2 inhibition—with fibroblast activation imaging as a pharmacodynamic readout.
6. Design antifibrotic trials with molecular enrichment: select on fibrotic activity, stratify on genotype, and use ECV or FAPI-PET change as the primary mechanistic endpoint.

The transition from compensated hypertrophy to fibrotic heart failure is not a single threshold event but a gradual, molecularly traceable process. The central clinical opportunity is to detect and interrupt it while the matrix remains remodellable.

## Figures and Tables

**Table 1:** Core signalling nodes in Ang II-induced cardiac fibrosis and their pharmacological tractability.

Node	Principal Effector Output	Strongest Supporting Evidence	Direction	Pharmacological Modulator (Status)
AT1R	Gq/PLC, MAPK, β-arrestin; stretch-activated	Human myocardial expression; mechanically activated signalling	Profibrotic	ARBs (approved); biased ligands (preclinical)
Chymase	ACE-independent Ang II generation	In vitro human cardiac membrane biochemistry [13]; in vivo evidence favours ACE [14]	Profibrotic (contested)	Chymase inhibitors (preclinical)
ACE2/Ang-(1-7)/Mas	Counter-regulatory peptide arm	ACE2-null mice show exaggerated Ang II remodelling	Antifibrotic	Recombinant ACE2, Mas agonists (early)
AT2R	NO/cGMP, phosphatase activation	Experimental organ protection	Antifibrotic	C21 (phase I–II, non-cardiac)
NOX2	Agonist-induced ROS	NADPH oxidase biology in failing heart	Profibrotic	No selective clinical agent
NOX4	Constitutive intracellular ROS	Conflicting gain- and loss-of-function data	Context-dependent	None (target validity unresolved)
NRF2/KEAP1	Antioxidant transcription	Attenuates experimental fibrosis	Antifibrotic	Bardoxolone-class (cardiac safety concerns)
CCR2^+^ macrophages	Monocyte-derived inflammation	Required for fibrosis in transverse aortic constriction [39]; not for fibrosis or diastolic dysfunction in an HFpEF model [40]	Context-dependent	CCR2 antagonists (preclinical)
NLRP3/IL-1β	Inflammasome activation	Mitochondria–inflammation circuit in HFpEF	Profibrotic	IL-1β blockade (approved, other indication)
IL-11	Obligate downstream of TGF-β in fibroblasts	Blockade prevents fibrosis without abolishing TGF-β	Profibrotic	Anti-IL-11 antibodies (preclinical/early)
TGF-β1–ALK5–SMAD2/3	Master fibrogenic transcription	Fibroblast-specific deletion abolishes fibrosis	Profibrotic	ALK5 inhibitors (toxicity-limited)
Latent TGF-β activation	Integrin/TSP-1-mediated	Integrin-dependent activation mechanism	Profibrotic	αv integrin inhibitors (trials in other organs)
MRTF-A/SRF	Cytoskeletal tension → transcription	Controls myofibroblast activation post-MI	Profibrotic	CCG-series inhibitors (preclinical)
YAP/TAZ	Stiffness sensing	Soft matrix suppresses fibroblast signature	Profibrotic	Verteporfin-class (preclinical)
TRPC6	Ca^2+^ entry → calcineurin	Required for myofibroblast transdifferentiation	Profibrotic	Selective blockers (preclinical)
PIEZO1	Stretch-activated Ca^2+^	Bidirectional effects reported	Context-dependent	Yoda1 (tool compound)
CCN2/CTGF	Autocrine amplification	Knockout does not prevent fibrosis (redundancy)	Profibrotic, non-obligatory	Pamrevlumab (non-cardiac trials)
Mineralocorticoid receptor	Direct fibroblast and immune activation	Aldosterone induces fibrosis directly	Profibrotic	MRAs incl. finerenone (approved)
Galectin-3	Macrophage-derived fibroblast activation	Mediator and biomarker	Profibrotic	Modified citrus pectin (preclinical)
LOX/LOXL2	Collagen crosslinking	LOXL2 inhibition reduces crosslinks and fibrosis	Profibrotic	SNT-5382, PXS-series (preclinical/early)
MMP/TIMP balance	Matrix turnover stoichiometry	Determines net accumulation	Bidirectional	Non-selective MMP inhibition failed clinically

**Table 2:** Molecular layers of inter-individual variation in Ang II-induced cardiac fibrosis: representative findings, the phenotype each layer assesses and the principal limitation of each.

Layer	Representative Findings	Phenotype Assessed	Principal Limitation (A, Addressable; I, Inherent)
RAAS candidate variants	*ACE* I/D, *AGT* M235T, *AGTR1* A1166C, *CYP11B2* −344C/T	Clinical HF, LV structure, BP	Small effects; inconsistent replication; population stratification (I effect size; A replication)
GWAS—heart failure	Multi-ancestry loci partitioned by HF subtype; MR-prioritised druggable proteins	Clinical HF and subtypes	Endpoint is downstream of fibrosis (A)
GWAS—fibrosis endophenotype	Loci for CMR native T1-derived interstitial fibrosis	Imaging-quantified fibrosis	Predominantly European-ancestry cohorts (A)
Rare variants	*FLNC* and *TTN* truncating variants; genotype-specific CMR signatures	Cardiomyopathy phenotype	Rare; limited to monogenic-enriched populations (I)
Polygenic risk scores	PRS predicts incident HF independent of clinical factors	Clinical HF	Poor cross-ancestry transportability (A)
Single-cell/nucleus transcriptomics	Fibroblast activation states; matrifibrocytes; CTHRC1^+^ cells	Cell-state composition	End-stage tissue bias; dissociation artefacts (I bias; A artefacts)
Spatial transcriptomics	Macrophage–fibroblast niches; spatial immune geography	Tissue architecture	Resolution/cost trade-off; few human Ang II-specific datasets (A)
DNA methylation	Cardiomyocyte methylation signatures in HF; *RASAL1* paradigm from kidney	Tissue and cell-free	Cardiac fibroblast methylation-lock unproven (A)
Histone/chromatin	BRD4-dependent enhancer remodelling; TEAD1–BRD4–Wnt4	Fibroblast activation	Pan-BET inhibition lacks cell selectivity (A)
Epitranscriptome	m^6^A control of *IL11* and *PIEZO2*	Fibroblast/macrophage transition	Early-stage; limited human data (A)
microRNA	miR-21 (pro), miR-29 (anti), miR-132, miR-34	Tissue and circulating	Cell-type-specific and opposing effects (I)
Long non-coding RNA	*Wisper*; LIPCAR	Tissue and circulating	Poor interspecies conservation (I)
Metabolomics	Serine–glycine/ATF4 axis; BCAA catabolism; acylcarnitines; TMAO	Cell and plasma	Diet, renal function and microbiome confounding (A design; I microbiome)
Proteomics	Plasma signatures for HF mortality and diastolic dysfunction	Plasma	Affinity-platform specificity; limited standardisation (I platform; A standards)

**Table 3:** Emerging biomarkers of Ang II-induced cardiac fibrosis, evaluated against tissue specificity, evidence level and clinical readiness.

Biomarker	Biological Meaning	Cardiac Specificity	Strongest Human Evidence	Key Confounders	Readiness
PICP/PIIINP	Collagen I/III synthesis	Low (systemic collagen)	Tracks biopsy fibrosis; responds to losartan	Bone, skin, liver turnover; renal clearance	Research/trial endpoint
CITP; CITP:MMP-1	Collagen I degradation; crosslinking inference	Low–moderate	Prognostic in HF and arrhythmia	Same as above	Research
MMP-2/9, TIMP-1	Matrix turnover balance	Low	Associated with hypertensive remodelling	Inflammation, malignancy	Research
Galectin-3	Macrophage-driven fibrogenesis	Low	Prognostic in HF	Renal function, systemic fibrosis	Clinically available; limited incremental value
Soluble ST2	IL-33/ST2 mechanical–inflammatory axis	Low	Independent prognosis (meta-analysis)	Inflammation; less affected by age/renal function	Clinically available
GDF-15	Generalised cellular stress	Very low	Consistent prognosis across cohorts	Renal, inflammatory, oncological	Prognostic adjunct
CCN2/CTGF	Fibrogenic amplification	Low	Correlates with fibrotic burden	Systemic fibrosis	Research
miR-21	Fibroblast ERK derepression	Moderate (fibroblast-enriched)	Elevated in failing myocardium	Normalisation, haemolysis	Research
miR-29 family	Direct collagen mRNA repression	Moderate	Downregulated peri-infarct	Opposing cell-type effects	Research
LIPCAR	Mitochondrial lncRNA, remodelling	Moderate	Predicts HF and mortality post-MI	Assay standardisation	Research
EV-encapsulated RNA	Cell-of-origin-attributable signalling	Potentially high	Mechanistic proof in models	Isolation methodology	Early research
cfDNA methylation deconvolution	Cell-type-specific cell death/turnover	Potentially very high	Heart-specific methylated marker described	Sensitivity at low fractions; cost	Early research
CMR native T1/ECV	Diffuse interstitial expansion (burden)	High	Biopsy-validated; prognostic meta-analysis	Field strength, sequence, haematocrit	Clinical; proposed surrogate endpoint
LGE	Focal replacement fibrosis	High	Prognostic in DCM	Insensitive to diffuse fibrosis	Clinical
^68^Ga-FAPI PET	Fibroblast activation (activity)	High	Concordant with histopathology in DCM	Availability, cost, radiation	Early clinical

## Data Availability

No new data were created or analyzed in this study. Data sharing is not applicable to this article.

## Supplementary Materials

The following supporting information can be downloaded at: https://www.mdpi.com/article/10.3390/ijms27219944/s1.

## Abbreviations

The following abbreviations are used in this manuscript:

ACE	angiotensin-converting enzyme
ACE2	angiotensin-converting enzyme 2
ADAM17	a disintegrin and metalloproteinase 17
AGT	angiotensinogen (gene)
ALK5	activin receptor-like kinase 5
Ang A	angiotensin A
Ang II	angiotensin II
Ang III	angiotensin III
Ang IV	angiotensin IV
AP-1	activator protein 1
ARB	angiotensin receptor blocker
AT1	angiotensin II type 1
AT1R	angiotensin II type 1 receptor
AT2R	angiotensin II type 2 receptor
BCAA	branched-chain amino acid
BET	bromodomain and extra-terminal
BP	blood pressure
BRD4	bromodomain-containing protein 4
CAD	coronary artery disease
CCL5	C-C chemokine ligand 5
CCN2	cellular communication network factor 2
CCR2	C-C chemokine receptor type 2
cfDNA	cell-free DNA
cGMP	cyclic guanosine monophosphate
CI	confidence interval
CITP	carboxy-terminal telopeptide of collagen type I
CKD	chronic kidney disease
CMR	cardiac magnetic resonance
COL3	collagen type III
CTGF	connective tissue growth factor
CTHRC1	collagen triple helix repeat containing 1
CV	cardiovascular
CYP11B2	aldosterone synthase (gene)
DCM	dilated cardiomyopathy
DEG	differentially expressed gene
DOI	digital object identifier
ECM	extracellular matrix
ECV	extracellular volume fraction
EF	ejection fraction
eGFR	estimated glomerular filtration rate
EndMT	endothelial-to-mesenchymal transition
ERK	extracellular signal-regulated kinase
EV	extracellular vesicle
FAP	fibroblast activation protein
FAPI	fibroblast activation protein inhibitor
GDF-15	growth differentiation factor 15
GRSF1	guanine-rich RNA sequence binding factor 1
GWAS	genome-wide association study
HF	heart failure
HFpEF	heart failure with preserved ejection fraction
HR	hazard ratio
ICM	ischaemic cardiomyopathy
IHD	ischaemic heart disease
IL	interleukin
JNK	c-Jun N-terminal kinase
JPH2	junctophilin-2
KEAP1	Kelch-like ECH-associated protein 1
LGE	late gadolinium enhancement
lncRNA	long non-coding RNA
LOX	lysyl oxidase
LOXL2	lysyl oxidase-like 2
LV	left ventricular
LVH	left ventricular hypertrophy
m6A	N6-methyladenosine
MACE	major adverse cardiovascular events
MAPK	mitogen-activated protein kinase
MeCP2	methyl-CpG binding protein 2
MI	myocardial infarction
MMP	matrix metalloproteinase
MR	mineralocorticoid receptor
MRA	mineralocorticoid receptor antagonist
MrgD	Mas-related G protein-coupled receptor D
MRTF-A	myocardin-related transcription factor A
NADPH	nicotinamide adenine dinucleotide phosphate
NFAT	nuclear factor of activated T cells
NF-κB	nuclear factor kappa B
NLRP3	NLR family pyrin domain containing 3
NOX	NADPH oxidase
NRF2	nuclear factor erythroid 2-related factor 2
NT-proBNP	N-terminal pro-B-type natriuretic peptide
OR	odds ratio
Orai1	calcium release-activated calcium channel protein 1
PET	positron emission tomography
PI3K	phosphoinositide 3-kinase
PICP	procollagen type I carboxy-terminal propeptide
PIEZO1	piezo-type mechanosensitive ion channel component 1
PIEZO2	piezo-type mechanosensitive ion channel component 2
PINP	procollagen type I amino-terminal propeptide
PIIINP	procollagen type III amino-terminal propeptide
PK	pharmacokinetics
PLC	phospholipase C
PRS	polygenic risk score
RAAS	renin–angiotensin–aldosterone system
ROS	reactive oxygen species
RUNX1	runt-related transcription factor 1
SGLT2	sodium–glucose co-transporter 2
snRNA-seq	single-nucleus RNA sequencing
SOCE	store-operated calcium entry
SOD2	superoxide dismutase 2
SRF	serum response factor
sST2	soluble suppression of tumorigenicity 2
STIM1	stromal interaction molecule 1
TAVI	transcatheter aortic valve implantation
TAZ	transcriptional co-activator with PDZ-binding motif
TEAD	TEA domain transcription factor
TET3	tet methylcytosine dioxygenase 3
TGF-β	transforming growth factor beta
TIMP	tissue inhibitor of metalloproteinase
TMAO	trimethylamine N-oxide
TRPC6	transient receptor potential cation channel subfamily C member 6
YAP	Yes-associated protein
YTHDF1	YTH domain-containing family protein 1
