# Supplementary material for: Angiotensin II-Induced Cardiac Fibrosis: Genomic and Molecular Drivers, Emerging Biomarkers and Therapeutic Perspectives
Source: Int J Mol Sci. 2026 Nov 6;27(21):9944. doi: 10.3390/ijms27219944 (PMC13763969; doi:10.3390/ijms27219944)
Supplement: Supplementary file 1 [file ijms-27-09944-s001.zip › ijms-4656309-supplementary.pdf]

Supplementary Materials

Angiotensin II-Induced Cardiac Fibrosis: Genomic and Molecular Drivers, Emerging Biomarkers and Therapeutic Perspectives

Nancy Lucero Martínez-Rodríguez, Javier Alonzo Campos-González, César López-Camarillo and Oscar Medina-Contreras

Manuscript ijms-4656309

Table S1. Search terms and query structure.

Databases: Scopus and PubMed/MEDLINE. Coverage: database inception to September 2026. In Scopus the terms below were combined as TITLE-ABS-KEY queries restricted to journal articles and reviews published in English; retrieved records were cross-checked against PubMed/MEDLINE. A further pass restricted to the two most recent publication years was performed in both indexes.

Keywords: “angiotensin II”, “renin–angiotensin–aldosterone system”, “AT1 receptor”, “cardiac fibrosis”, “myocardial fibrosis”, “cardiac fibroblast”, “myofibroblast”, “extracellular matrix”, “collagen turnover”, “adverse cardiac remodelling”, “heart failure”, “oxidative stress”, “NADPH oxidase”, “TGF-β1”, “mechanotransduction”, “genome-wide association study”, “polygenic risk score”, “DNA methylation”, “single-cell RNA sequencing”, “spatial transcriptomics”, “microRNA”, “long non-coding RNA”, “m6A”, “metabolomics”, “fibrosis biomarker”, “galectin-3”, “soluble ST2”, “GDF-15”, “NT-proBNP”, “PICP”, “PIIINP”, “extracellular volume”, “late gadolinium enhancement”, “FAPI-PET” and “antifibrotic therapy”, cross-referenced against named genes (AGT, AGTR1, ACE, CYP11B2, TGFB1, NOX4, IL11, LOXL2, FAP) and against therapeutic classes (angiotensin-converting enzyme inhibitors, angiotensin-receptor blockers, mineralocorticoid-receptor antagonists, sodium–glucose cotransporter 2 inhibitors). In Scopus these terms were combined as TITLE-ABS-KEY queries restricted to journal articles and reviews published in English and the retrieved records were cross-checked against PubMed/MEDLINE. A further pass restricted to the two most recent publication years was performed in both indexes to capture recent primary work.

Table S2. In vitro and in vivo model studies establishing the mechanisms discussed in Sections 3–5 and the therapeutic targets of Section 7.2. Abbreviations: AAV9, adeno-associated virus serotype 9; BCAA, branched-chain amino acid; MMT, macrophage-to-myofibroblast transition; ROS, reactive oxygen species; snRNA-seq, single-nucleus RNA sequencing. Other abbreviations are defined in the abbreviation list.

Model system	Species / preparation	Perturbation	Readout	Principal finding	Ref.
Ang II infusion, pressure-dissociated design	Rodent in vivo	Chronic Ang II infusion ± BP control	Fibroblast proliferation (BrdU)	Ang II stimulates perivascular and interstitial fibroblast proliferation independently of blood pressure and aldosterone, though full dissociation is difficult to achieve experimentally	[10]
Post-MI and Ang II infusion models	Rat in vivo (ARB treatment) [17]; mouse, ACE2 knockout [18]	ACE2–Ang-(1-7)–Mas augmentation or ACE2 deletion	Remodelling and fibrosis	Counter-regulatory arm is a determinant, not a bystander: ACE2 loss exaggerates the Ang II response	[17,18]

Model system	Species / preparation	Perturbation	Readout	Principal finding	Ref.
Diabetic cardiomyopathy model	Mouse in vivo, cardiomyocyte-specific deletion	*Adam17* deletion	LV remodelling	Cardiomyocyte ADAM17 deletion ameliorates remodelling	[21]
Gain- and loss-of-function under load	Mouse in vivo (cardiomyocyte, endothelial)	NOX4 overexpression or deletion	Hypertrophy and fibrosis	NOX4 effects are context-dependent: NOX4 overexpression is profibrotic in one model [32], whereas cardiomyocyte-targeted overexpression was protective and deletion worsened remodelling via impaired angiogenesis [33,34]	[32,33,34]
Pressure overload / MMT model	Mouse in vivo; macrophages	ALKBH5-dependent m^6A of *Il11*	Macrophage-to-myofibroblast transition	Epitranscriptomic control of IL-11 drives MMT and pathological fibrosis	[46]
Ang II infusion	Mouse in vivo, *Ccl5* knockout	CCL5 deletion	Fibrosis, hypertrophy, LV function	Platelet-derived CCL5 deletion attenuates fibrosis with reduced M2 polarisation	[49]
Pressure overload; obese diabetic model	Mouse in vivo, fibroblast-specific deletion	Loss of TGF-β–SMAD2/3 in fibroblasts	Interstitial fibrosis; LV function	Fibroblast TGF-β signalling is necessary and cell-autonomous for fibrosis	[61,62]
Chronic pressure overload	Mouse in vivo, cardiac *Ctgf* knockout	CTGF deletion	Hypertrophy and fibrosis	CTGF is dispensable under chronic load: redundancy in the amplification layer	[74]
Mechanotransduction model	Mouse in vivo; cardiac fibroblasts	YTHDF1-dependent m^6A of *PIEZO2* mRNA	Cardiac fibrosis	m^6A modification of a mechanosensor transcript modulates the fibrotic response	[69]
Pharmacological demethylation in renal fibrosis	Mouse in vivo; fibroblasts in vitro	*Low-dose hydralazine; RASAL1 promoter demethylation*	Fibroblast activation; fibrosis	Methylation-dependent silencing sustains activation and is pharmacologically reversible	[138]
Endogenous reversal of promoter hypermethylation	Mouse in vivo, experimental kidney fibrosis	*BMP7 administration; Tet3-mediated hydroxymethylation*	Rasal1 promoter methylation; fibrosis	Antifibrotic effect of BMP7 associates with normalisation of Rasal1 hypermethylation through Tet3	[137]
Fibrosis model with fibroblast-specific deletion	Mouse in vivo; cardiac fibroblasts	*Ythdf1* deletion; *Runx1* silencing	Cytoskeletal remodelling; fibrosis	m^6A state of *RUNX1*, not transcript abundance, sets transcription-factor output	[143]
Redox–epigenome coupling	Mouse in vivo; cardiac fibroblasts	MeCP2 repression of SOD2; fibroblast SOD2 deficiency	Mitochondrial ROS; ferroptosis	MeCP2 represses SOD2, and it is the loss of SOD2 that raises mitochondrial ROS and drives cytoskeletal remodelling and ferroptosis; MeCP2 knockdown rescues SOD2 and mitigates these changes	[144]
Diet–genotype interaction	Transgenic mice, human *AGT* Hap-I / Hap-II	High-salt diet	*AGT* promoter methylation and expression	Salt alters methylation and raises human *AGT* expression in a haplotype-dependent manner	[145]
miR-21 gain/loss of function	Mouse in vivo; cardiac fibroblasts	miR-21 silencing; Ang II/osteopontin axis	ERK–MAPK signalling; interstitial fibrosis	miR-21 derepresses ERK–MAPK via *SPRY1*; silencing attenuates fibrosis and dysfunction	[147]

Model system	Species / preparation	Perturbation	Readout	Principal finding	Ref.
Therapeutic miR-21 silencing	Mouse in vivo, chronic Trypanosoma cruzi infection; cardiac fibroblasts	LNA-anti-miR-21	Cardiac fibrosis; inflammatory response	Silencing reduces cardiac fibrosis in a non-Ang II model; no miR-21–SPRY1 relationship detected in vivo	[148]
Ang II/phenylephrine infusion with AAV9-shRNA; ^{13}C tracing	Mouse in vivo; human cardiac fibroblasts	ACLY knockdown	Fibrotic area; histone acetylation	De novo lipogenesis supplies acetyl-CoA for histone acetylation at fibrogenic loci	[169]
YAP activation model with human snRNA-seq comparison	Mouse in vivo; cardiac fibroblasts	Fibroblast YAP activation	Glycolysis; fibroinflammation	YAP-driven glycolysis promotes fibroinflammation and disrupts fibroblast identity, with chamber specificity	[170]
Loss-of-function in cardiomyocytes	Mouse in vivo	GRSF1 deletion	BCAA homeostasis; HF phenotype	Post-transcriptional BCAA control links the metabolomic signature to HF pathogenesis	[171]
Longitudinal FAPI-PET with infusion withdrawal	Mouse in vivo	Ang II/phenylephrine infusion, then withdrawal	Myocardial [^{68}Ga]FAPI-46 uptake	Signal tracks FAP-positive to FAP-negative transition: a dynamic, not cumulative, measure	[203]
Adoptive cell therapy	Mouse in vivo	FAP-directed CAR T cells	Established fibrosis	Activated fibroblasts can be eliminated and established fibrosis reversed (CAR T given 1 week after Ang II/phenylephrine initiation, once fibrosis was established; fibrosis reduced in 7 of 7 mice and nearly eliminated in 5 of 7, with the effect persisting at 8 weeks)	[226]
Post-MI local delivery	Mouse in vivo	IL-11-blocking antibody ± ROS-responsive hydrogel	Scar fibrosis; tissue stiffness; contractility	Free antibody modestly effective; hydrogel encapsulation prolongs retention and markedly improves efficacy	[230]
Pharmacological BET inhibition	Mouse in vivo; fibroblasts and macrophages	BET inhibitor	Fibroblast activation; macrophage polarisation	Attenuates activation and polarisation, but pan-BET inhibition lacks cell selectivity	[139,141]
Post hoc analysis of a randomised trial	Patients with recent acute coronary syndrome and type 2 diabetes	Apabetalone	Major adverse cardiovascular events	Clinical signal with a BET inhibitor in patients, stratified by a liver fibrosis score	[142]
Direct cardiac reprogramming	Mouse in vivo; resident fibroblasts	Reprogramming to induced cardiomyocytes	Fibroblast state; fibrosis; contractile function	About 2% of resident fibroblasts converted to induced cardiomyocytes; profibrotic fibroblasts shifted to a quiescent antifibrotic state with suppression of Meox1	[231]
Galectin-3 inhibition	Rodent in vivo	Modified citrus pectin and related inhibitors	Experimental fibrosis	Reduces fibrosis, supporting galectin-3 as a tractable matricellular target	[232]
Diabetic cardiomyopathy model	Mouse in vivo, type 2 diabetic KK-Ay	Empagliflozin	Oxidative stress; cardiac fibrosis	Glycaemic control with empagliflozin attenuated myocardial oxidative stress	[222]

Model system	Species / preparation	Perturbation	Readout	Principal finding	Ref.
				and fibrosis, through inhibition of TGF-β/Smad and activation of Nrf2/ARE	
Pressure overload / DOCA model	Mouse in vivo, including SGLT2 knockout	Empagliflozin; NHE1 inhibition with cariporide	NHE1–nitric oxide pathway; fibrosis; systolic and diastolic function	Protection against fibrosis and dysfunction is preserved in SGLT2 knockout mice and mimicked by cariporide, supporting an NHE1–NO mechanism independent of SGLT2	[223]
Relaxin-based approaches	Rodent in vivo	Relaxin / RXFP1 agonism	Experimental fibrosis	Preclinical support persists despite the neutral phase 3 experience in acute HF	[234]
